# Microsatellite expansion redirects chromatin modifiers to confer insect resistance to Bt bioinsecticides

**DOI:** 10.1093/nar/gkag1058

**Published:** 2026-10-27

**Authors:** Chunzheng Ouyang, Kaiyue Sun, Fu Wang, Fan Ye, Liuhong Zhu, Lina Dong, Yang Bai, Zhouqiang Cheng, Mingmei Wu, Neil Crickmore, Xuguo Zhou, Youjun Zhang, Zhaojiang Guo

**Affiliations:** State Key Laboratory of Vegetable Biobreeding, Department of Plant Protection, Institute of Vegetables and Flowers, Chinese Academy of Agricultural Sciences, Beijing 100081, China; State Key Laboratory of Vegetable Biobreeding, Department of Plant Protection, Institute of Vegetables and Flowers, Chinese Academy of Agricultural Sciences, Beijing 100081, China; State Key Laboratory of Vegetable Biobreeding, Department of Plant Protection, Institute of Vegetables and Flowers, Chinese Academy of Agricultural Sciences, Beijing 100081, China; State Key Laboratory of Vegetable Biobreeding, Department of Plant Protection, Institute of Vegetables and Flowers, Chinese Academy of Agricultural Sciences, Beijing 100081, China; State Key Laboratory of Vegetable Biobreeding, Department of Plant Protection, Institute of Vegetables and Flowers, Chinese Academy of Agricultural Sciences, Beijing 100081, China; State Key Laboratory of Vegetable Biobreeding, Department of Plant Protection, Institute of Vegetables and Flowers, Chinese Academy of Agricultural Sciences, Beijing 100081, China; State Key Laboratory of Vegetable Biobreeding, Department of Plant Protection, Institute of Vegetables and Flowers, Chinese Academy of Agricultural Sciences, Beijing 100081, China; State Key Laboratory of Vegetable Biobreeding, Department of Plant Protection, Institute of Vegetables and Flowers, Chinese Academy of Agricultural Sciences, Beijing 100081, China; State Key Laboratory of Vegetable Biobreeding, Department of Plant Protection, Institute of Vegetables and Flowers, Chinese Academy of Agricultural Sciences, Beijing 100081, China; School of Life Sciences, University of Sussex, Brighton BN1 9QE, United Kingdom; Department of Entomology, School of Integrative Biology, College of Liberal Arts & Sciences, University of Illinois Urbana-Champaign, Illinois 61801-3795, United States; State Key Laboratory of Vegetable Biobreeding, Department of Plant Protection, Institute of Vegetables and Flowers, Chinese Academy of Agricultural Sciences, Beijing 100081, China; State Key Laboratory of Vegetable Biobreeding, Department of Plant Protection, Institute of Vegetables and Flowers, Chinese Academy of Agricultural Sciences, Beijing 100081, China

## Abstract

Microsatellites are highly mutable genomic elements, yet their role in adaptive gene regulation in insects remains poorly understood. Here, we show that a tetranucleotide (ATGT) microsatellite expansion in the promoter of *ABCC2*, a key *Bacillus thuringiensis* (Bt) toxin receptor gene, epigenetically silences its expression and confers resistance to Bt Cry1Ac toxin in *Plutella xylostella* (L.). Mechanistically, long repeats (≥27 units) in Cry1Ac-resistant strains preferentially recruit the histone methyltransferase E(z), promoting H3K27me2/3 deposition, chromatin compaction, and transcriptional silencing, whereas short repeats in susceptible strains maintain open chromatin through Tip60-mediated H4K5 acetylation. This repeat expansion alone is sufficient to repress *ABCC2*, thereby conferring Bt resistance, while repeat length, chromatin state, and associated phenotypes partially revert under relaxed selection. These findings define a previously uncharacterized *cis*-acting element dynamically orchestrating insecticide resistance-associated gene expression and identify microsatellite-driven chromatin remodeling as a general and plastic regulatory strategy underlying rapid xenobiotic-resistance evolution in insects.

## Graphical Abstract

**Graphical Abstract:**
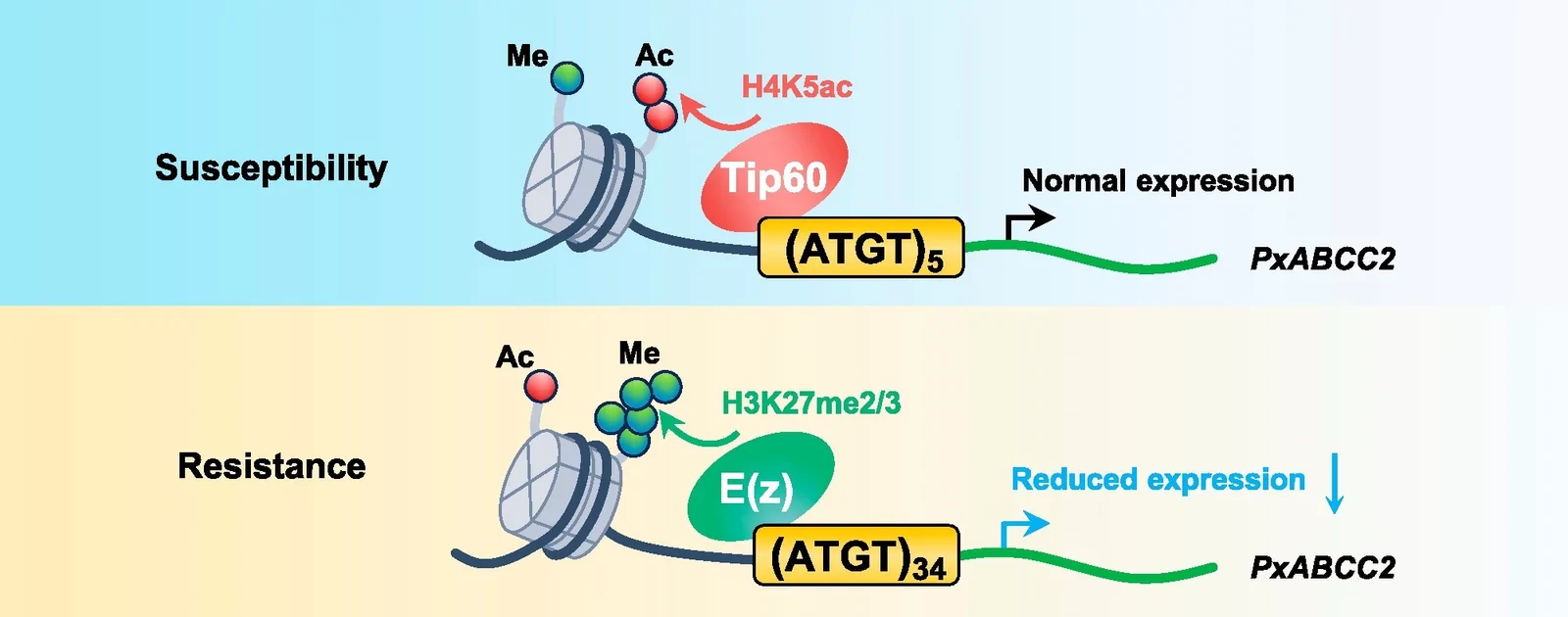

## Introduction

The insect pathogenic bacterium *Bacillus thuringiensis* (Bt) can produce a diverse array of pesticidal proteins that can kill various insect pests, primarily from Lepidoptera, Coleoptera, and Diptera, with high specificity and environmental safety [1]. The subsequently developed Bt-based foliar sprays and genetically engineered crops have become a cornerstone of modern agricultural biotechnology [2, 3]. However, the durability of these Bt biotechnology products is seriously jeopardized due to the widespread resistance evolution in target insect pests [4, 5]. To date, practical resistance to Bt sprays and Bt crops has been confirmed in at least 13 insect species worldwide [2, 6]. This pressing challenge underscores the urgent need to unravel the molecular mechanisms underpinning Bt resistance to inform sustainable resistance management strategies and safeguard the long-term utility of Bt biotechnology [7, 8].

The insecticidal activity of Bt toxins relies on a multi-step process that targets the larval midgut: following ingestion, protoxins are proteolytically activated, allowing the mature toxin to bind to specific midgut receptors; this binding facilitates subsequent toxin oligomerization and pore formation, leading to cellular lysis, midgut damage, and insect death [9]. Several classes of midgut membrane proteins function as primary Bt toxin receptors, including cadherin-like proteins (CAD), aminopeptidase N (APN), alkaline phosphatase (ALP), and ATP-binding cassette (ABC) transporters [10, 11]. Among them, ABC transporters, particularly ABCC2, have been widely recognized as the cardinal midgut receptor for Bt Cry1 toxins, and *cis*-acting mutations or *trans*-regulatory repression of ABCC2 have frequently been implicated in high-level Cry1 resistance across diverse lepidopteran insects [12, 13].

The diamondback moth, *Plutella xylostella* (L.), is a global notorious agricultural pest of cruciferous crops, causing annual economic losses of 4–5 billion USD [14]. It was the first documented insect species to evolve high-level resistance to Bt biopesticides under field conditions and serves as a model insect for interrogating the molecular basis of Bt resistance [15]. Our earlier studies have substantiated that ABCC2 is the most important midgut receptor for the Bt Cry1Ac toxin in *P. xylostella* [16–18], and further revealed its reduced expression conferred high-level Cry1Ac resistance in *P. xylostella*, which is modulated by the MAPK-directed *trans*-regulatory mechanism [19–28]. Nonetheless, the existence of potential *cis*-regulatory variations in the *ABCC2* promoter and their contribution to its stable downregulation remain unexplored.

Tandem repeats (TRs) are genomic sequences composed of contiguous head-to-tail repetitions of short nucleotide motifs, typically categorized by unit length into microsatellites (1–6 bp), minisatellites (6–100 bp), and megasatellites (>100 bp) [29]. Beyond motif size, TRs are principally characterized by extensive variation in repeat number, predominantly driven by the insertion or deletion of entire repeat units through replication slippage and homologous recombination [30, 31]. Microsatellites, or short tandem repeats (STRs), are especially abundant and highly polymorphic, making them widely used as genetic markers [32]. Microsatellites are ubiquitously distributed across both coding and non-coding regions, including promoters, untranslated regions, and introns [33, 34]. Initially discovered through reassociation kinetics in the 1960s, their functional significance was overlooked for decades until emerging evidence, primarily from mammalian and plant systems, implicated microsatellites in gene regulation and genome plasticity [34–36]. However, their functional relevance in insects, especially in the regulation of stress- or resistance-associated genes, remains poorly characterized.

In this study, we identify an ATGT microsatellite in the *ABCC2* promoter that undergoes repeat expansion in resistant *P. xylostella* strains. We show that variation in repeat length selectively biases the recruitment of distinct histone-modifying enzymes, promoting a shift from an acetylated, transcriptionally permissive state to a methylated, repressive chromatin configuration that silences *ABCC2* expression and confers Bt Cry1Ac resistance in *P. xylostella*. This work expands the regulatory landscape of microsatellites and deepens our understanding of Bt resistance evolution in insects.

## Materials and methods

### Insect strains and cell lines

A total of eight *P. xylostella* strains were initially used in this study, all of which have been described before [23, 37, 38]. Four Bt Cry1Ac-susceptible strains were employed: DBM1Ac-S, collected from experimental fields in New York, USA, in 1988; and SY-S, NK-S, and YQ-S, all collected from experimental fields in Beijing, China, and named according to the initials of their respective collection regions. Four Bt Cry1Ac-resistant strains were also used: DBM1Ac-R, a field-evolved Bt-resistant strain (>1600-fold) collected in 1992 from Florida, USA; NIL-R, a near-isogenic Bt Cry1Ac-resistant strain developed in our lab through successive backcrosses and Bt Cry1Ac selection (seven times) between DBM1Ac-S and DBM1Ac-R strains [39, 40]; and SZ-R and SH-R, which were collected in Shenzhen and Shanghai, China, and further selected by Bt Cry1Ac protoxin and Bt var. *kurstaki* (Btk) formulations in our lab, respectively. Compared to the susceptible DBM1Ac-S strain, these resistant strains exhibited 4500-fold, 5200-fold, 700-fold, and 1400-fold resistance to Bt toxins or Btk formulations, respectively. In addition, a new gene-edited strain named S-(ATGT)_34_ was constructed as part of the work. Detailed information for all strains is provided (Supplementary Tables S1–S3). All strains were reared under controlled temperature and humidity conditions in dedicated insect-rearing chambers. Adults were fed a honey-water solution, while larvae were reared on live cabbage leaves of the “Jingfeng No. 1” cultivar (*Brassica oleracea var. capitata*).

Three cell lines were used in this study. *Drosophila melanogaster* S2 cells were cultured in SFX medium supplemented with penicillin and streptomycin. *Spodoptera frugiperda* Sf9 cells were maintained in SF900-III medium supplemented with both antibiotics and fetal bovine serum (FBS). Both insect cell lines were cultured as adherent monolayers at 27°C in non-CO_2_ incubators. *Homo sapiens* HEK293T cells were cultured in Dulbecco’s modified Eagle’s medium supplemented with penicillin, streptomycin, and FBS at 37°C in a humidified incubator with 5% CO_2_.

### Toxin preparation and bioassay

Cry1Ac protoxin was purified from the Btk strain HD-73 and quantified using the Bradford protein assay, with bovine serum albumin as the standard [41]. To evaluate the toxicity of Cry1Ac protoxin, a 72-h leaf-dip bioassay was conducted using third-instar larvae of *P. xylostella*. Seven concentrations of Cry1Ac were prepared, and for each concentration, 10 larvae were tested. Each concentration treatment was performed in four independent biological replicates. Mortality data were analyzed using Polo Plus version 2.0 (LeOra Software) based on probit analysis to estimate the median lethal concentration (LC_50_) and its 95% confidence limits (CLs). Non-overlapping 95% CLs between LC_50_ values were considered indicative of statistically significant differences.

### gDNA isolation, RNA extraction, and gene cloning

Fourth-instar larvae of *P. xylostella* were dissected in ice-cold insect Ringer’s solution (0.5 mM KCl, 130 mM NaCl, 0.1 mM CaCl_2_) to collect various tissue samples. Total RNA was extracted using TRIzol reagent (Invitrogen) following the manufacturer’s protocol. For quantitative polymerase chain reaction (qPCR) analysis, first-strand complementary DNA (cDNA) was synthesized using the PrimeScript RT reagent kit (TaKaRa). For gene cloning purposes, cDNA was synthesized using the PrimeScript II first Strand cDNA Synthesis Kit (TaKaRa) according to the manufacturer’s instructions.

Genomic DNA (gDNA) for promoter cloning was extracted using the TIANamp Genomic DNA Kit (TIANGEN). DNA used for genotyping was obtained by lysis using the TransDirect Animal Tissue PCR Kit (TransGen).

The coding sequences (CDSs) of all genes cloned in this study (GenBank accession nos. PV959442–PV959452) were identified and corrected by BLASTn alignment based on our previously established *P. xylostella* transcriptome database [42]. Specific primers for each gene were designed using Primer Premier 5.0 software (Premier Biosoft) (Supplementary Table S5). PCR amplification was performed on an S1000 Thermal Cycler (Bio-Rad) using the PrimeSTAR Max DNA Polymerase Kit (TaKaRa) under the following conditions: initial denaturation at 98°C for 2 min; 35 cycles of 98°C for 30 s, 56°C for 15 s, and 72°C for 1 min; followed by a final extension at 72°C for 10 min. PCR products were purified using the Wizard SV Gel and PCR Cleanup System (Promega) and subsequently subcloned into the pEASY-T1 vector (TransGen) for sequencing.

### Phylogenetic and bioinformatic analyses

Promoter sequences of *ABCC2* cloned from individual *P. xylostella* strains were used for phylogenetic analysis. Sequence alignments were performed using ClustalW in MEGA X (http://www.megasoftware.net/), and unrooted maximum likelihood phylogenetic trees were constructed based on 1000 bootstrap replicates. Trees were visualized and edited using iTOL (https://itol.embl.de/). For bioinformatic analyses, sequence assembly and alignment were performed in DNAMAN 9.0 (Lynnon BioSoft). CDS sequences were corrected with TBtools (https://www.tbtools.com/). Protein CDSs were translated using the ExPASy Translate Tool (https://web.expasy.org/translate/), and predicted molecular weight (Mw) and isoelectric point (pI) were computed using Compute pI/Mw (http://web.expasy.org/compute_pi/). TR elements and microsatellite regions were identified using CENSOR (https://www.girinst.org/censor/).

### Plasmid construction and dual-luciferase reporter assay

The *ABCC2* promoter regions were amplified from gDNA of various *P. xylostella* strains using PCR with specific primers (Supplementary Table S5). PrimerSTAR Max DNA Polymerase (TaKaRa) was used for high-fidelity amplification. Primer design was based on the *P. xylostella* genome information available in the Lepbase (http://ensembl.lepbase.org/Plutella_xylostella_pacbiov1/) and the updated genome in GenBank (https://www.ncbi.nlm.nih.gov/bioproject/PRJEB34571/). Full-length promoter sequences were subsequently used as templates to generate a series of truncated or mutated promoter constructs using specific primers (Supplementary Table S5). Mutant and combination promoter variants were generated using the M5 Site-Directed Mutagenesis Kit (Mei5 Biotechnology) following the manufacturer’s instructions. All promoter fragments were cloned into the pGL4.10 firefly luciferase reporter vector (Promega) between the KpnI and BglII restriction sites to construct various promoter-pGL4.10 reporter plasmids for luciferase activity analysis. CDSs of transcription factors (TFs) or interacting proteins were cloned into the pAc5.1/V5-His B vector (Invitrogen) between the KpnI and XhoI restriction sites to generate effector constructs. Primer sequences are provided (Supplementary Table S5). Dual-luciferase reporter assays were conducted as previously described [25, 43]. In brief, to assess promoter and microsatellite activity, S2/293T cells were cotransfected with 600 ng of promoter-pGL4.10/(ATGT)_n_-pGL4.23 and 200 ng of pGL4.73 plasmids. To evaluate the TFs, 200 ng of (ATGT)_n_-pGL4.23 and 100 ng of pGL4.73 were cotransfected together with 600 ng of pAC-TFs recombinant plasmids. Transfections were carried out using Lipofectamine 2000/3000 (Invitrogen) according to the manufacturer’s instructions. At 48 h post-transfection, cells were lysed, and luciferase activity was measured using the Dual-Luciferase Reporter Assay System (Promega) following the recommended protocol. The Renilla luciferase gene under the control of the SV40 promoter in the pGL4.73 vector served as an internal control.

### Immunofluorescence assay

Reconstructed Pie-EGFP-N1 plasmids—with either (ATGT)_5_ or (ATGT)_34_ inserted upstream of the EGFP—were transfected into Sf9 cells using FuGENE HD transfection reagent (Promega) at a 1:3 (plasmid:reagent, v/v) ratio. Non-transfected cells were used as negative controls, and cells transfected with empty Pie-EGFP-N1 vector served as positive controls. At 48 h post-transfection, cells were fixed in 4% pre-chilled paraformaldehyde (w/v) for 15 min, followed by permeabilization with 0.5% Triton X-100 for 20 min. After washing three times with phosphate buffered saline (PBS), nuclei were stained with 5 μM Hoechst (Abcam) for 30 min at room temperature in the dark. Cells were washed again three times with PBS, mounted using glycerol, and imaged using a Zeiss LSM 980 confocal laser scanning microscope for fluorescence analysis.

### Microsatellite copy number monitoring

PCR was performed using gDNA as a template in a 25 μl reaction volume with 35 cycles under the following conditions: initial denaturation at 94°C for 30 s; annealing at an optimized temperature between 50°C and 60°C (depending on the primer pair) for 45 s; and extension at 72°C for 1–4 min based on the expected product size. A final extension step was conducted at 68°C for 5 min using a high-fidelity polymerase with a high-GC content buffer (TaKaRa). PCR products were purified, subcloned into pEASY-T1 vector (TransGen), and Sanger sequenced to determine copy numbers of the ATGT repeats in individual insects.

### CRISPR/Cas9 experiment

The CRISPR/Cas9 tool [27, 44] was employed to insert the 34 ATGT repeats into the *ABCC2* promoter region of the susceptible *P. xylostella* strain DBM1Ac-S. Two single-guide RNAs (sgRNA1 and sgRNA2) targeting upstream and downstream sequences flanking the desired insertion site were designed using the CRISPR RGEN tool Cas-Designer (http://www.rgenome.net/cas-designer/). The sgRNA templates were synthesized (Supplementary Table S5), transcribed *in vitro* using the MEGAshortscript T7 Transcription Kit (Ambion), and purified with the MEGAclear Kit (Ambion). A 497-nt single-stranded oligodeoxynucleotide (ssODN) containing the full 34 ATGT repeats flanked by homology arms was synthesized as donor DNA. A mixture of sgRNAs (150 ng/μl), Cas9 protein (100 ng/μl; Thermo Fisher Scientific), and ssODN (1 μg/μl) was injected into 543 freshly laid pre-blastoderm eggs of DBM1Ac-S. Of these, 226 larvae hatched (41%), and 125 (55% of hatched) developed into adults (G_0_). Fifty male and fifty female G_0_ moths were randomly selected for sibling crosses to generate the G_1_ progeny. A total of 118 G_1_ pupae were collected and genotyped using specific primers flanking the insertion site. Four genotypes were identified: wild type (23%), CRISPR/Cas9-induced non-homologous end joining (29%), heterozygous for (ATGT)_n_ insertion (36%), and homozygous for the (ATGT)_34_ insertion (12%). Six male and eight female homozygous G_1_ moths carrying the (ATGT)_34_ insertion were intercrossed to establish a homozygous S-(ATGT)_34_ strain. Subsequent sequencing of 22 G_2_ individuals confirmed stable homozygosity for the (ATGT)_34_ insertion at the *ABCC2* promoter.

### Quantitative real-time PCR analysis

qPCR reactions were performed using a QuantStudio 3 Real-Time PCR System (Applied Biosystems) with the 2.5 × SYBR Green MasterMix Kit (TIANGEN), as previously described [45, 46]. A standard two-step amplification protocol was used: initial denaturation at 95°C for 1 min, followed by 40 cycles of 95°C for 5 s and 57°C–60°C for 15 s, depending on the specific primers. The expression levels of *ABCC2* and candidate histone-modifying enzymes were quantified using the 2^−ΔΔCt^ method. The ribosomal protein L32 gene (*RPL32*, GenBank accession no. AB180441) was used as the internal reference. Melt curve analysis was performed to ensure amplification specificity. Each sample was tested with three biological replicates and four technical replicates. Primer sequences are provided (Supplementary Table S5). Statistical significance was evaluated using one-way analysis of variance (ANOVA) followed by Tukey’s post hoc test.

### Protein extraction and western blotting

Midgut tissues were collected from fourth-instar larvae of *P. xylostella* strains (50 larvae per group). Tissues were homogenized in ice-cold lysis buffer (Sigma–Aldrich) supplemented with protease and phosphatase inhibitors (Roche), followed by two rounds of 5-min sonication. Lysates were centrifuged at 4°C for 10 min, and the supernatant was collected for protein quantification using the Bradford assay (Biomed). Equal amounts of protein were mixed with loading buffer (CWBIO), denatured by boiling, and separated by 8% sodium dodecyl sulfate (SDS)–polyacrylamide gel electrophoresis. Proteins were then transferred onto PVDF membranes (Merck Millipore), which were blocked with skim milk and sequentially incubated with primary antibody against ABCC2 (1:3000; AtaGenix) overnight at 4°C and secondary HRP-conjugated anti-rabbit antibody (1:5000; CWBIO) at room temperature for 1 h. Signals were detected using SuperSignal West Pico chemiluminescent substrate (Thermo Fisher Scientific) and imaged with a Tanon-5200 chemiluminescence system (Tanon). Band intensity was quantified using ImageJ version 1.51.

### DNA pull-down and mass spectrometry analysis

Biotin-labeled primers targeting the (ATGT)_n_ region were commercially synthesized (General Biosystems), with biotin modifications at the 5′ end. The target DNA probe was amplified by PCR and purified using standard protocols. Midgut tissues were freshly dissected from fourth-instar larvae of *P. xylostella* and minced into small fragments on ice. Tissues were homogenized in precooled cell lysis buffer using a mechanical homogenizer. The homogenate was centrifuged, and the resulting supernatant (protein extract) was collected and kept on ice for subsequent steps. DNA pull-down assays were performed by incubating the biotin-labeled DNA probes with streptavidin-conjugated magnetic beads at 4°C overnight to ensure strong DNA-bead coupling. These beads were then incubated with the midgut lysates overnight at 4°C to allow protein–DNA binding. After incubation, the supernatant was discarded, and the beads were washed several times with wash buffer to eliminate unbound and nonspecifically bound proteins. Bound proteins were eluted by boiling the beads in SDS sample buffer, which released DNA-associated proteins from the magnetic beads. The eluted protein samples were then subjected to liquid chromatography-tandem mass spectrometry (LC-MS/MS) analysis for protein identification.

### Heterologous expression and protein purification

Heterologous expression and protein purification were performed as previously described [19] with minor modifications. Briefly, the Tip60 and E(z) proteins were expressed using the Bac-to-Bac baculovirus expression system (Invitrogen) according to the manufacturer’s instructions. The CDSs of *PxTip60* and *PxE(z)* were synthesized and individually cloned into the pFastBacHT B vector to generate the recombinant pFastBacHT B constructs.

The resulting plasmids were transformed into *Escherichia coli* DH10Bac competent cells, and positive bacmid clones were selected and transfected into Sf9 cells (Invitrogen). The P1 viral stocks were harvested 72 h after transfection, and expression of the recombinant proteins was confirmed by western blot analysis. To increase viral titers, P2 viral stocks were generated by infecting Sf9 cells with the P1 stocks. Sf9 cells were collected 72 h after infection with the P2 viral stocks, and recombinant Tip60 and E(z) proteins were purified from cell lysates using a His-tag Protein Purification Kit (Beyotime Biotechnology) following the manufacturer’s protocol.

### Yeast one-hybrid assay

To identify proteins interacting with the (ATGT)_n_ region, the (ATGT)_5/34_ sequences were separately cloned into the pAbAi vector to generate bait constructs. These constructs were then integrated into the yeast one-hybrid (Y1H) Gold yeast strain following the Matchmaker Gold Yeast One-Hybrid System (Clontech) [47, 48]. The minimum inhibitory concentration of aureobasidin A (AbA) required to suppress autoactivation was empirically determined for each bait strain to ensure optimal growth conditions.

For large-scale screening, a pre-constructed *P. xylostella* cDNA yeast library was co-transformed with the (ATGT)_34_ bait strain. Putative positive clones were identified based on growth on SD/-Leu medium supplemented with AbA and further validated by secondary screening using selective antibiotic-containing media. To perform targeted Y1H interaction assays, CDSs of candidate TFs or histone-modifying enzymes were cloned into the pGADT7 prey vector and transformed into bait yeast strains. Positive interactions were assessed by growth on SD/-Leu plates containing AbA. Yeast co-transformed with pGADT7-p53 and pAbAi-p53 was used as a positive control, while yeast transformed with empty pGADT7 and bait plasmids served as a negative control.

### Electrophoretic mobility shift assay

Recombinant Tip60 and E(z) proteins were heterologously expressed in an insect cell (Sf9) expression system and purified using a His-tag Protein Purification Kit (Beyotime Biotechnology) according to the manufacturer’s instructions. Double-stranded oligonucleotide probes corresponding to the (ATGT)_5/34_ microsatellite sequences were synthesized and labeled with biotin at the 5′-terminus (General Biosystems). Prior to electrophoresis, biotin-labeled DNA probes were incubated with the purified recombinant proteins at 25°C for 30 min to allow DNA–protein complex formation. The reaction mixtures were then subjected to non-denaturing polyacrylamide gel electrophoresis. Following electrophoresis, DNA–protein complexes were electro-transferred onto nylon membranes and detected using the LightShift Chemiluminescent EMSA Kit (Thermo Fisher Scientific) following the manufacturer’s protocol. Chemiluminescent signals were visualized using a Tanon-5200 imaging system (Tanon).

### ChIP-qPCR assay

Chromatin immunoprecipitation (ChIP) assays was conducted using the EZ-Magna ChIP A/G Chromatin Immunoprecipitation Kit (Merck Millipore) following the manufacturer’s instructions. Midgut tissues were dissected from fourth-instar larvae in ice-cold 1 × PBS supplemented with 1 mM phenylmethylsulfonyl fluoride (PMSF; Beyotime Biotechnology) and finely minced using a scalpel. Tissues were cross-linked in 1% formaldehyde for 15 min at room temperature, and the reaction was quenched with 0.125 M glycine for 5 min. Tissue homogenization was performed on ice using a mechanical tissue grinder (Sigma–Aldrich) to release nuclei. Chromatin was extracted and sonicated to yield DNA fragments averaging ∼600 bp in length. Immunoprecipitation was performed overnight at 4°C using 5 μg of antibody [(Pan-Kac (anti-acetyllysine, PTM-101, PTM Biolabs), Pan-Kme2 (anti-di-methyllysine, PTM-606, PTM Biolabs), Pan-Kme3 (anti-tri-methyllysine, PTM-601, PTM Biolabs), H3K27me (PTM-5110, PTM Biolabs), H3K27me2 (PTM-5312, PTM Biolabs), H3K27me3 (PTM-5002, PTM Biolabs), H4K5ac (PTM-163, PTM Biolabs), H4K8ac (PTM-164, PTM Biolabs), or H4K16ac (PTM-122, PTM Biolabs), as appropriate] pre-bound to protein A/G agarose beads. An anti-IgG antibody (5 μg; Sigma–Aldrich) served as the negative control. DNA was purified from the immunoprecipitates and analyzed by qPCR using primers (Supplementary Table S5). The specificity of the antibodies used for chromatin profiling was validated by immunoblotting (Supplementary Fig. S8), and the strain-specific primer sequences used to amplify the corresponding promoter regions in the susceptible and resistant strains are provided (Supplementary Table S4 and Supplementary Fig. S9).

### CUT&Tag-qPCR assay

Cleavage under targets and tagmentation (CUT&Tag) assays were performed using the Hyperactive Universal CUT&Tag Assay Kit for qPCR (Vazyme) following the manufacturer’s instructions. Briefly, midgut tissues were dissected from *P. xylostella* larvae and gently homogenized to obtain nuclei. Approximately 1 × 10^5^ nuclei were immobilized on ConA Beads Pro. Bead-bound nuclei were incubated overnight at 4°C with the indicated primary antibodies, with normal rabbit IgG included as a negative control. After washing, samples were incubated with a secondary antibody (1:100 dilution) at room temperature for 1 h with gentle rotation. pA/G-Tnp Pro was then added and incubated for an additional 1 h at room temperature to allow enzyme binding. Tagmentation was initiated by adding 1 × tagmentation buffer L (TTBL; Vazyme) and incubating the reaction at 37°C for 60 min. DNA spike-in was subsequently added, and samples were incubated at 55°C for 10 min to release tagmented DNA fragments. DNA was purified using the kit-provided reagents according to the manufacturer’s protocol. Enrichment of histone modifications at the *ABCC2* promoter was quantified by qPCR using primers designed to amplify the microsatellite-containing region of the promoter.

### Chromatin accessibility analysis

Chromatin accessibility analysis was performed using the Chromatin Accessibility Assay Kit (Abcam) following the manufacturer’s protocol. Midgut tissues were dissected from fourth-instar larvae in ice-cold PBS supplemented with 1 mM PMSF (Beyotime Biotechnology), minced, and homogenized in pre-chilled cell lysis buffer to release nuclei. After centrifugation, the nuclear pellet was washed and resuspended in 50 μl of transposition reaction mix to ensure efficient contact with transposase. Following the transposition reaction, DNA was purified using a DNA purification kit, and the eluted DNA was analyzed by qPCR with specific primers (Supplementary Table S5).

### RNA interference and inhibitor injection

RNA interference (RNAi) treatments were performed as previously described [49, 50], with minor modifications, as summarized below. All designed small interfering RNA (siRNA) sequences were subjected to BLAST analysis against the GenBank database (https://www.ncbi.nlm.nih.gov/) and the *P. xylostella* genome resources, Lepbase (http://ensembl.lepbase.org/Plutella_xylostella_pacbiov1/), to ensure target specificity and avoid off-target effects. Chemically synthesized siRNAs were commercially synthesized (GenePharma). Each gene-specific siRNA carried a 3′ dTdT overhang and incorporated 2′-O-methyl-modified cytidine (C) and uridine (U) residues to enhance stability and reduce immune responses. A scrambled siRNA sequence (siNC) was used as a negative control. Detailed siRNA sequences are provided (Supplementary Table S5).

siRNAs were dissolved in Injection Buffer (Merck Millipore) and mixed with an equal volume of Metafectene Pro transfection reagent (Biontex) to facilitate cellular uptake. Using a FemtoJet 4i and InjectMan 4 Microinjection System (Eppendorf), 70 nl of the siRNA formulation, containing a final siRNA concentration of 25 µM, was injected into newly molted third-instar larvae of *P. xylostella*. Larvae injected with the corresponding vehicle formulation or scrambled siRNA (siNC) served as controls. Total RNA was extracted 48 h post-injection, and knockdown efficiency was evaluated by qPCR.

Inhibitor treatment was performed using the same microinjection procedure. A-395 and NU9056 were initially dissolved in DMSO at 10 mg/ml and diluted to a final concentration of 1 mg/ml in a delivery formulation containing Injection Buffer and Metafectene Pro at a 1:1 (v/v) ratio. Each newly molted third-instar larva was injected with 70 nl of the inhibitor formulation, corresponding to 70 ng inhibitor per larva. Control larvae received an equivalent volume of the corresponding vehicle formulation containing the same concentration of DMSO but without inhibitor. Samples were collected 48 h after injection for subsequent analyses.

### Selection and relaxation experiments

To monitor changes in *ABCC2* promoter microsatellite composition under different selection regimes, the susceptible strain DBM1Ac-S and its near-isogenic Cry1Ac-resistant strain NIL-R were used. For the selection assay, DBM1Ac-S larvae were subjected to continuous Cry1Ac exposure for 15 consecutive generations. In each generation, larvae were treated with a toxin concentration of 2 mg/l (LC_90_ value), imposing strong selection pressure with high mortality. Surviving insects were reared to adulthood and used to establish the next generation under the same selection regime. For the relaxed-selection assay, NIL-R was maintained for 15 consecutive generations under standard rearing conditions without Bt exposure or resistance selection. At each generation, 100 individuals were randomly sampled for microsatellite genotyping. Repeat copy number at the ATGT-containing region of the *ABCC2* promoter was determined for each individual separately by PCR amplification of the repeat-containing fragment followed by sequence verification. In parallel, *PxABCC2* expression and histone modification status at the repeat-containing promoter region were monitored in the same generation from the same population using independent parallel samples. For these analyses, fourth-instar larvae were collected at each generation. Total RNA was extracted from larval midgut tissues, and *ABCC2* transcript levels were quantified by qPCR. Histone modification status at the repeat-containing promoter region was assessed by ChIP-qPCR, and fold enrichment of H4K5ac and H3K27me3 was calculated relative to IgG controls. Changes in repeat composition, gene expression, and histone modification levels were compared across generations to evaluate the stability or partial reversion of microsatellite-associated chromatin states under selection and relaxed-selection conditions.

### Statistical analyses and data visualization

Statistical analyses were performed using IBM SPSS Statistics v23.0, except for concentration–mortality analyses, which were conducted using PoloPlus v2.0. Continuous molecular data were analyzed using one-way ANOVA followed by Tukey’s multiple-comparison test. For qPCR, four technical replicates were averaged for each biological replicate, and statistical analyses were performed using ΔCt values. Larval survival and mortality were analyzed using binomial generalized linear models based on the numbers of dead and surviving larvae. Each treatment included three biological replicates of 30 larvae. Pairwise comparisons were adjusted using the Holm method. Fifteen-generation mortality data were analyzed using binomial generalized linear models with generation as a continuous predictor. Concentration–mortality data were analyzed by probit regression. LC_50_ values, 95% CLs, slopes, and resistance ratios were calculated. Data are presented as mean ± SEM unless otherwise stated. In figures, ns indicates *P* ≥ .05, whereas *, **, and *** indicate *P* < .05, *P* < .01, and *P* < .001, respectively. Figures were prepared using GraphPad Prism v8.3, SigmaPlot v12.5, and Microsoft Office 2010.

## Results

### Identification of potential *cis*-mutations in the *ABCC2* promoter

Previously, we demonstrated that downregulation of *ABCC2* is causally associated with Cry1Ac resistance in *P. xylostella* [26]. This downregulation was found to be mediated by the *trans*-acting TF called FTZ-F1 [24]. We had also found that expression of two other different downregulated receptors (ABCG1 and mALP) was further reduced by *cis*-acting mutations [25, 48]. To investigate whether *cis*-acting regulatory elements contribute to reduced *ABCC2* expression in resistant strains, we cloned ~2 kb of the genomic region upstream of the *ABCC2* CDS. Using a dual-luciferase system, we established that expression from the promoter derived from a resistant strain was significantly lower than that from a susceptible strain’s promoter (Fig. 1A). To understand the basis of this differential activity, we then sequenced the promoter regions from multiple Cry1Ac-susceptible and -resistant strains (Supplementary Tables S1 and S2). Phylogenetic analysis of the obtained sequences displayed four distinct haplotypes (Fig. 1B). A dual-luciferase assay performed with representative samples (S1, S3, R1, and R5) from each haplotype indicated higher activity from the promoters derived from the susceptible strains (Fig. 1C), suggesting the presence of functional *cis*-regulatory variations contributing to decreased *ABCC2* expression in resistant strains.

**Figure 1.**
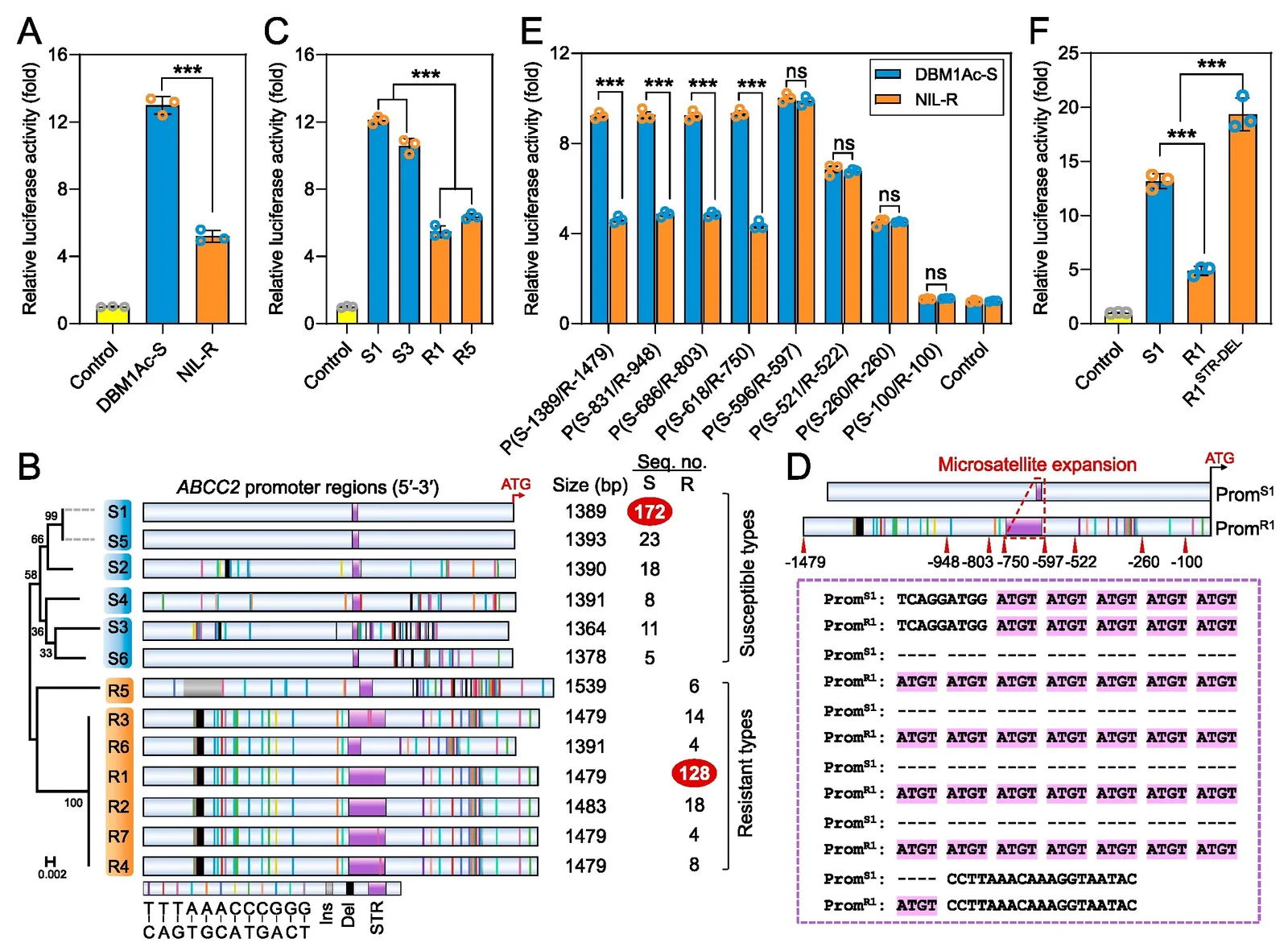
Microsatellite expansion in the *ABCC2* promoter of Bt Cry1Ac-resistant *P. xylostella* strains. (**A**) Relative luciferase activity of the promoters from the DBM1Ac-S and NIL-R strains. (**B**) Characterization of *ABCC2* promoter variants cloned from DBM1Ac-S (susceptible) and NIL-R (resistant) strains. Thirteen representative promoters from DBM1Ac-S are labeled S1–S6, and five from NIL-R are labeled R1–R7. Promoter lengths and the relative abundance of each sequence type are shown on the right. The most abundant susceptible-derived (S1) and resistant-derived (R1) promoters are highlighted. Nucleotide variations relative to S1 are indicated by colored vertical lines; insertions and deletions are shown with gray and black boxes, respectively. The STR or microsatellite and its expanded region are marked with purple boxes. The phylogenetic tree on the left was constructed using the maximum likelihood method based on the optimized Tamura 3-parameter (T92) model from 1378 aligned nucleotide positions, with bootstrap values shown from 1000 replicates. (**C**) Relative luciferase activity of representative susceptible (S1, S3) and resistant (R1, R5) promoters. (**D**) Schematic of ATGT repeat expansion. The purple box marks the core ATGT repeat region, and red arrows indicate deletion endpoints corresponding to panel E. The expanded repeat region is highlighted. (**E**) Progressive 5′–3′ deletions of major *ABCC2* promoter types to identify functional regulatory elements associated with expression differences. (**F**) Functional analysis of ATGT repeat expansion on promoter activity. “STR-DEL” indicates complete deletion of the 136-bp STR element from the promoter. In panels (A, C, E, and F), S2 cells were cotransfected with pGL4.10 constructs containing various *ABCC2* promoter fragments fused to the firefly luciferase gene, and the pGL4.73 Renilla luciferase vector driven by the SV40 early promoter as an internal control. Relative luciferase activity was calculated as the ratio of firefly to Renilla signals, normalized to the activity of the empty pGL4.10 vector. Data represent means ± SEM from three independent biological replicates (*n* = 3). Statistical significance was determined by one-way ANOVA followed by Tukey’s multiple comparisons test: ns, not significant, ***P < .001.

A notable feature of the promoter sequences was the presence of a polymorphic tetranucleotide (ATGT) TR region (Fig. 1D and Supplementary Fig. S1). To initially assess whether this microsatellite array might be affecting expression, we created a number of deletions in the S1 and R1 promoters, choosing end points based on the edges of the TRs and on other sequence differences. The results clearly showed that in deletions that went beyond the microsatellite region (between −750 and −597 bp), the differential activity between S1 and R1 was lost (Fig. 1E). To assess the role of the repeat region more directly, just this region was deleted from the R1 promoter, and as can be seen from the result (Fig. 1F), expression levels were increased, strongly indicating that the microsatellite array was repressing expression.

### Microsatellite expansion mediates the downregulation of *ABCC2*

To further interrogate the role of the microsatellite in *PxABCC2* downregulation, we quantified the repeat copy number in susceptible and resistant strains. The result showed that all the tested susceptible strains carried a relatively low number of ATGT repeats (predominantly 5), whereas the resistant strains (except SZ-R) uniformly harbored a high number of repeats (predominantly 34) (Fig. 2A and Supplementary Tables S1 and S2). To assess the intrinsic regulatory effect of the repeat element, we cloned either 5 or 34 repeats of ATGT upstream of a minimal promoter in the pGL4.23 luciferase vector and transfected the constructs into insect (S2) and human (HEK293T) cells. Luciferase assays revealed that the 34-repeat construct exerted pronounced transcriptional repression in both cell types, with a dose-dependent effect observed as plasmid concentration increased. In contrast, the 5-repeat construct exhibited o nly mild transcriptional repression, which remained largely unchanged across plasmid doses (Supplementary Fig. S2A). Given the natural variation in ATGT repeat number among different individuals, we further analyzed constructs containing naturally occurring repeat lengths. This analysis showed that repeat numbers in the range of 31–35 conferred significantly stronger transcriptional repression than those with 3–27 repeats; moreover, no striking differences in expression were exhibited between the three longest or the three shortest constructs, implying a saturation effect (Fig. 2B). Finer-scale analysis within the 27–34 range confirmed a positive correlation between repeat number and transcriptional suppression, suggesting that transcriptional repression intensifies with increasing ATGT repeat expansion (Fig. 2C). These observations mirrored the pattern of full promoter activity from susceptible and resistant strains, supporting a causal link between ATGT repeat expansion and transcriptional repression of *PxABCC2*. This trend was consistent in both S2 and 293T cells, suggesting that the repressive function of microsatellite ATGT is both repeat number-dependent and evolutionarily conserved (Supplementary Fig. S2B).

**Figure 2.**
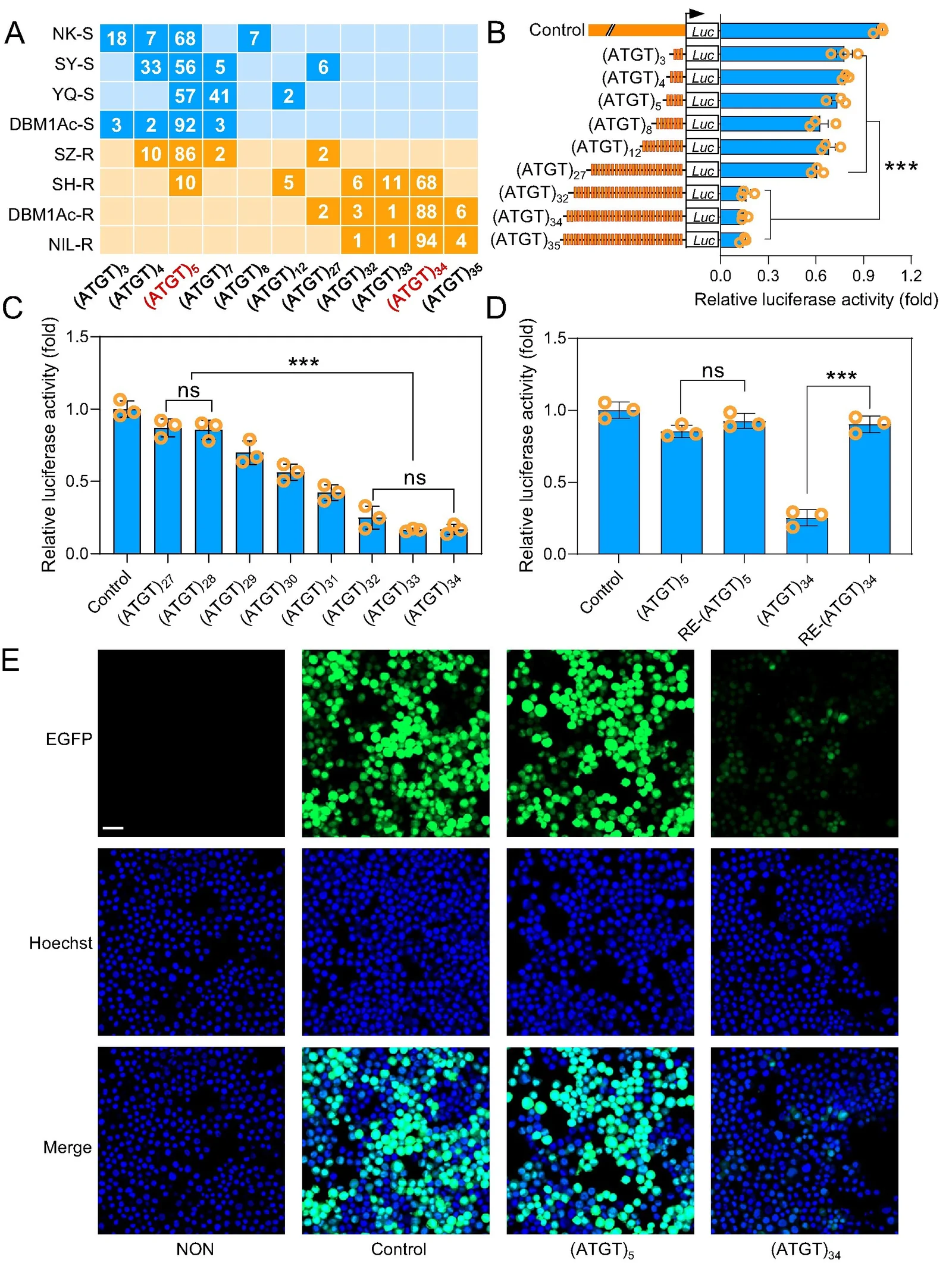
Microsatellite expansion confers transcriptional repression. (**A**) Distribution of ATGT repeat numbers among different susceptible and resistant *P. xylostella* strains. gDNA was extracted from 100 fourth-instar larvae each strain to determine repeat numbers. Strains highlighted with blue indicate susceptible strains; those with orange indicate resistant strains. (**B**) Transcriptional activity of naturally occurring ATGT repeat numbers in various *P. xylostella* strains. (**C**) Effects of artificially constructed repeat copy numbers on transcriptional activity. (**D**) Impact of repeat orientation on transcriptional activity. Different repeat numbers of ATGT were inserted upstream of a minimal promoter in the pGL4.23 firefly luciferase reporter vector. These constructs were cotransfected with pGL4.73 Renilla luciferase plasmid driven by the SV40 early promoter into S2 and 293T cells. Relative luciferase activity was calculated as the ratio of firefly to Renilla luciferase signals and normalized to the activity of the empty pGL4.23 vector. Data represent means ± SEM from three independent biological replicates (*n* = 3). Statistical significance was determined using one-way ANOVA followed by Tukey’s post hoc test: ns, not significant, ***P < .001. (**E**) Microsatellite-mediated regulation of cellular fluorescence intensity. Different ATGT repeats configurations were inserted upstream of the EGFP gene in the recombinant pie-EGFP plasmid and transfected into Sf9 cells. Green fluorescence represents EGFP expression; nuclei were counterstained with Hoechst and appear blue (Scale bar, 50 μm). Fluorescence was imaged and compared across constructs; the empty vector and un-transfected Sf9 cells served as positive and negative controls, respectively.

To test whether the sequence orientation of the microsatellite affected transcriptional regulation, we generated reverse-orientation (ATGT)_5/34_ constructs. These constructs failed to repress transcription, indicating that transcriptional suppression is dependent on the native orientation of the repeat and independent of copy number alone (Fig. 2D). To directly assess whether ATGT repeat expansion could suppress expression of an unrelated coding gene, we inserted either 5 or 34 repeats upstream of the EGFP CDS and transiently transfected Sf9 cells. Fluorescence imaging showed a marked reduction in EGFP expression in the 34-repeat group compared to the 5-repeat control (Fig. 2E), confirming that high-copy repeats can broadly repress transcription beyond the native ABCC2 context. These results established microsatellite ATGT as a dose-dependent, orientation-specific transcriptional inhibitor of insect genes.

### Microsatellite expansion is associated with a Cry1Ac resistance trait

To directly test the functional contribution of the microsatellite ATGT to Bt resistance, we employed CRISPR/Cas9-mediated genome editing to replace the microsatellite region from the susceptible strain with its counterpart from the resistant strain. Specifically, the 5-copy repeat in a susceptible background was replaced with a 34-copy repeat using a dual-sgRNA strategy and a single-stranded DNA donor template. The resulting edited strain, carrying the expanded repeat in the original susceptible genetic background, was designated as S-(ATGT)_34_ (Fig. 3A and Supplementary Table S3). To exclude potential confounding effects from sgRNA site removal and to assess the role of the expanded microsatellite in promoter function, we conducted luciferase reporter assays to compare the edited and original promoters. The results showed that introduction of the 34-copy repeat into the S1-type promoter markedly reduced its activity, whereas the sgRNA site deletion alone had no significant impact on promoter strength (Fig. 3B). Quantitative analysis of *ABCC2* expression at both messenger RNA (mRNA) and protein levels confirmed a substantial downregulation in the S-(ATGT)_34_ strain relative to the wild-type susceptible background (Fig. 3C and D). Notably, expression levels of other receptor (mALP, APN1, APN3a, ABCB1, ABCC3, ABCG1) and non-receptor (APN5, APN6, ABCC1) genes remained unchanged (Fig. 3D). In addition, immunofluorescence staining of fourth-instar larval midgut tissues further confirmed reduced ABCC2 protein levels in the S-(ATGT)_34_ strain (Fig. 3E), consistent with the observed transcriptional suppression. Functionally, this reduction in *ABCC2* expression translated into a dramatic increase in Cry1Ac resistance: the S-(ATGT)_34_ strain exhibited ~300-fold higher resistance compared to the parental susceptible line (Fig. 3F), a value similar in magnitude to that seen with an ABCC2 knockout [16].

**Figure 3.**
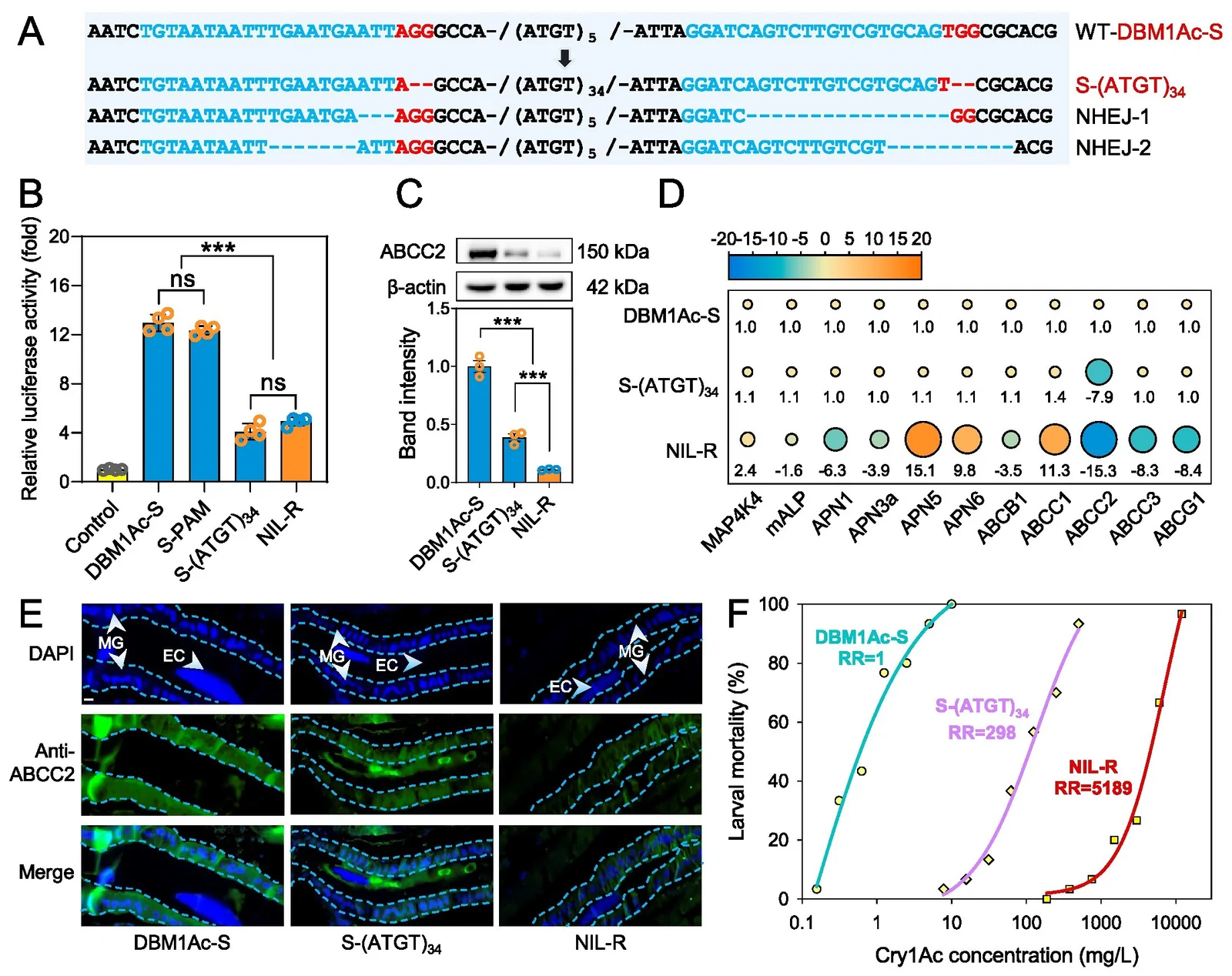
Expansion of microsatellite repeats mediates Cry1Ac resistance in *P. xylostella*. (**A**) Representative CRISPR/Cas9-induced mutations identified in G_1_ larvae using dual-sgRNA strategy, validated by single-colony PCR amplification and sequencing. (**B**) Promoter activity analysis of the genome-edited strain S-(ATGT)_34_, using the same dual-luciferase assay strategy as in Fig. 1A. (**C**) Protein expression levels of ABCC2 in DBM1Ac-S (susceptible), S-(ATGT)_34_ (genome-edited), and NIL-R (resistant) strains, measured by western blot. (**D**) Transcriptional levels of *ABCC2* and other midgut-related genes in the homozygous edited strain S-(ATGT)_34_. Transcript levels are represented as heatmap color blocks, normalized to each corresponding gene expression in the DBM1Ac-S strain (set as 1.0). Values greater than or equal to 1 are presented as positive fold increases, whereas values below 1 were converted to the negative reciprocal (−1/relative expression) to indicate the magnitude of downregulation. (**E**) Immunofluorescence detection of ABCC2 protein abundance in the midgut tissue of live larvae from DBM1Ac-S, S-(ATGT)_34_, and NIL-R strains. The location of the midgut is outlined by the dotted line (Scale bar, 50 μm). MG, midgut; EC, enteric cavity. (**F**) Log-dose response curves of DBM1Ac-S, S-(ATGT)_34_, and NIL-R strains after exposure to Cry1Ac protoxin. Data are presented as mean values ± SEM, *n* = 4 (B) and *n* = 3 (C), one-way ANOVA with Tukey’s test was used for comparison, ***P < .001. Concentration–mortality data in panel (F) were analyzed by probit regression.

### Microsatellite expansion rewires protein recruitment at the *ABCC2* promoter

Repetitive elements within gene regulatory regions can serve as scaffolds for nucleic acid-binding proteins, thereby influencing gene expression [51]. To investigate whether the *ABCC2* ATGT repeats interact with nuclear protein expression, we performed Y1H screening using bait constructs containing either 5 or 34 repeats. Unfortunately, the construct containing five repeats exhibited strong self-activation, precluding downstream screening (Supplementary Fig. S3). We therefore employed a cDNA pull-down assay using biotin-labeled probes to enrich interacting proteins from midgut extracts. Y1H screening using the (ATGT)_34_ bait identified 77 candidate proteins, while pull-down/MS analysis identified 246 candidates also by the same bait, with 16 proteins found in both datasets (Fig. 4A and Supplementary Tables S6 and S7). Of these 16 proteins, 9 were not found in the (ATGT)_5_ pull-down experiment. Among the overlapping proteins, we identified 9 TFs, one histone-modifying enzyme, and several others involved in chromatin structure or gene regulation. Because the pull-down/MS experiment was exploratory and used probes corresponding to different repeat lengths, it was not used to quantify binding affinity. Candidate interactions were therefore examined in subsequent one-to-one validation assays.

**Figure 4.**
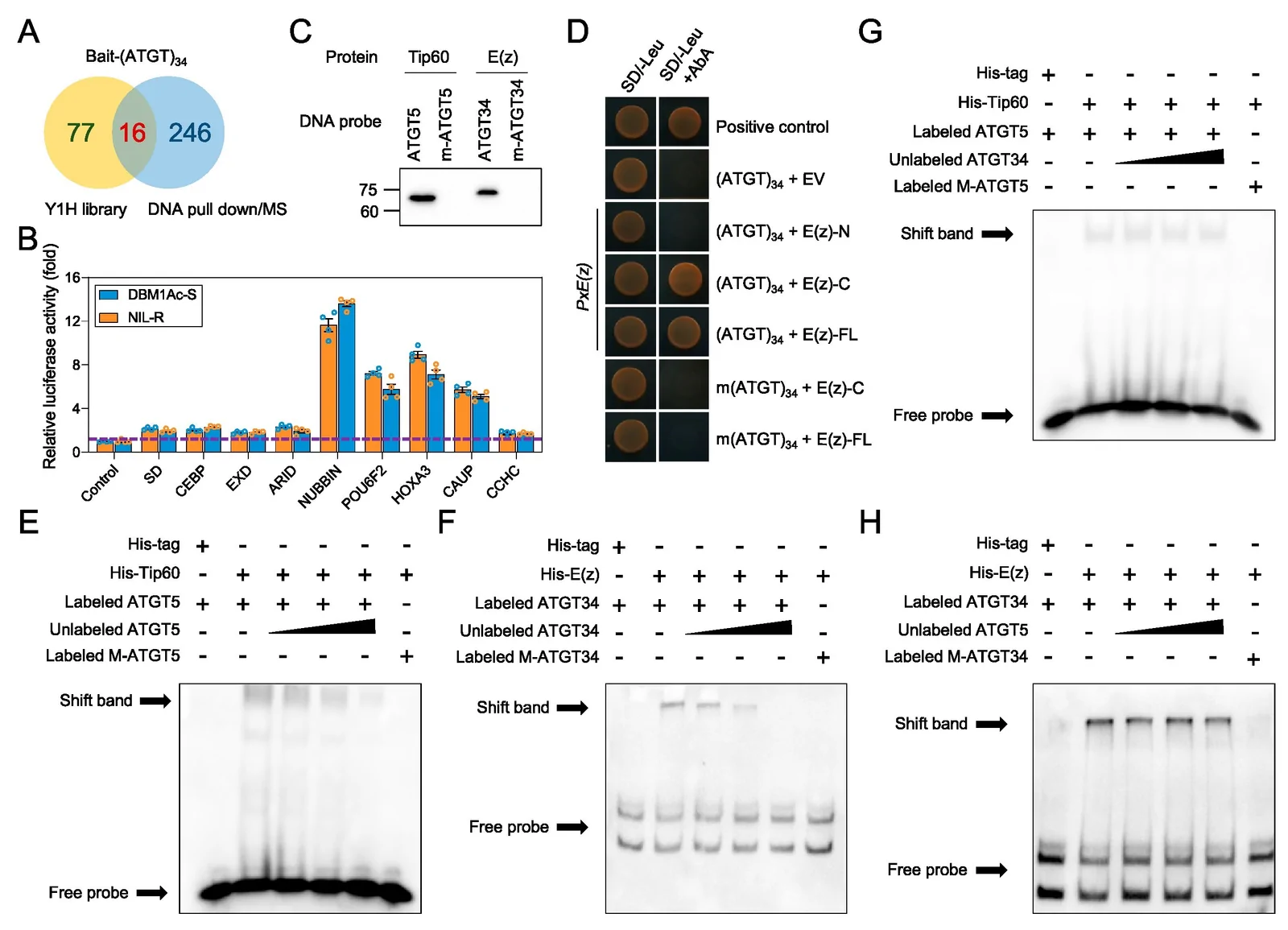
Microsatellite function as a nuclear protein–binding platform. (**A**) The Y1H library screening and DNA pull-down/MS databases were employed to seek potential protein binding to the (ATGT)_34_. (**B**) Effects of 9 TFs on the expression of the ATGT repeat-containing *ABCC2* gene. Each pAc5.1-TF expression vector was co-transfected with a (ATGT)_5/34_-pGL4.23 reporter plasmid into S2 cells to detect luciferase activity. An empty pAc5.1 vector was used as a control. The relative luciferase activity (fold) was calculated based on the value of the control, which was assigned an arbitrary value of 1. (**C**) DNA pull-down assays examining the association of Tip60 and E(z) with ATGT repeats of different lengths. (**D**) Y1H assay to assess binding of (ATGT)_34_ to E(z). Full-length E(z), N-terminal fragment, and C-terminal fragment used in Y1H assays. Electrophoretic mobility shift assays (EMSAs) measuring the direct binding of Tip60 to the short (ATGT)_5_ repeat (**E**) and of E(z) to the expanded (ATGT)_34_ repeat using purified recombinant proteins (**F**). EMSA assays showing repeat length-specific and non-interchangeable binding of Tip60 and E(z) to ATGT microsatellite. Tip60 binds the short (ATGT)_5_ probe and is not competed by the expanded repeat (**G**), whereas E(z) selectively binds the (ATGT)_34_ probe and is not displaced by the short repeat (**H**). The wedge indicates increasing amounts of unlabeled competitor DNA.

To explore the functional relevance of the TFs identified, we co-transfected each with reporter constructs containing either (ATGT)_5_ or (ATGT)_34_ in insect cells. None of these TFs exhibited repressive activity; instead, most reporter activities were enhanced to some degree (Fig. 4B), suggesting that transcriptional repression may not be directly mediated by these factors.

Notably, several histone-related proteins and chromatin-associated factors were identified in these screens. Among them, the acetyltransferase Tip60 was detected exclusively in the (ATGT)_5_ pull-down, whereas the methyltransferase E(z) was identified in both the Y1H screen and the (ATGT)_34_ pull-down, but not in the (ATGT)_5_ pull-down (Fig. 4C and D; Supplementary Fig. S4), suggesting the possibility that ATGT repeats might regulate *ABCC2* transcription through epigenetic mechanisms. We first performed a one-to-one Y1H assay to validate the interaction between E(z) and the expanded repeat. Full-length E(z) interacted with the (ATGT)_34_ bait. A C-terminal fragment containing the predicted CXC zinc-binding region also interacted with the bait, whereas the N-terminal fragment did not (Fig. 4D and Supplementary Fig. S5A). These results independently supported the E(z)–(ATGT)_34_ association and localized the interaction to a region containing the CXC module.

To directly test whether repeat length preferentially associates with of histone-modifying enzymes, we expressed *PxTip60* and *PxE(z)* in an insect cell system (Sf9) and purified the recombinant proteins for biochemical assays (Supplementary Fig. S6). DNA pull-down experiments using purified proteins demonstrated that Tip60 preferentially associated with the (ATGT)_5_ repeat, whereas E(z) was selectively enriched by the expanded (ATGT)_34_ repeat (Fig. 4C). These interactions were further examined by EMSA using purified proteins and both repeat-length alleles. Tip60 produced a detectable mobility shift with the (ATGT)_5_ probe but not with the (ATGT)_34_ probe, whereas E(z) produced a detectable shift with the (ATGT)_34_ probe but not with the (ATGT)_5_ probe under the conditions tested (Fig. 4E and F). Moreover, Tip60 did not produce detectable shifts with either the length-matched scrambled probe or an equal-length probe composed of a different short-repeat sequence. Similarly, E(z) did not produce detectable shifts with either the length-matched scrambled probe or an equal-length probe composed of a different expanded-repeat sequence (Supplementary Fig. S7). These controls indicate that the observed associations were dependent on both ATGT sequence composition and repeat length rather than probe length or repetitive character alone. In cross-competition assays, excess unlabeled long-repeat probes failed to disrupt Tip60 binding to the (ATGT)_5_ repeats (Fig. 4G), and conversely, binding of E(z) to the expanded (ATGT)_34_ repeats was not competed by the short-repeat probe (Fig. 4H). These results indicated that Tip60 and E(z) recognize microsatellite repeats in a length-specific and non-interchangeable manner. Collectively, these findings demonstrated that microsatellite expansion rewires the protein recruitment landscape at the *ABCC2* promoter, establishing a repeat length-dependent platform that selectively engages distinct chromatin-modifying enzymes.

### Microsatellite expansion alters histone modifications and chromatin configuration

Given the preferential association of histone-modifying enzymes with the ATGT repeats, we next examined whether repeat expansion correlates with changes in histone modification status at the *ABCC2* promoter. Chromatin immunoprecipitation followed by qPCR (ChIP-qPCR) revealed pronounced differences in histone modification patterns between susceptible and resistant strains. Specifically, resistant strains carrying expanded ATGT repeats exhibited elevated levels of histone di- and trimethylation and reduced histone acetylation within the ATGT-containing promoter region, whereas susceptible strains with shorter repeats displayed the opposite trend (Fig. 5A).

**Figure 5.**
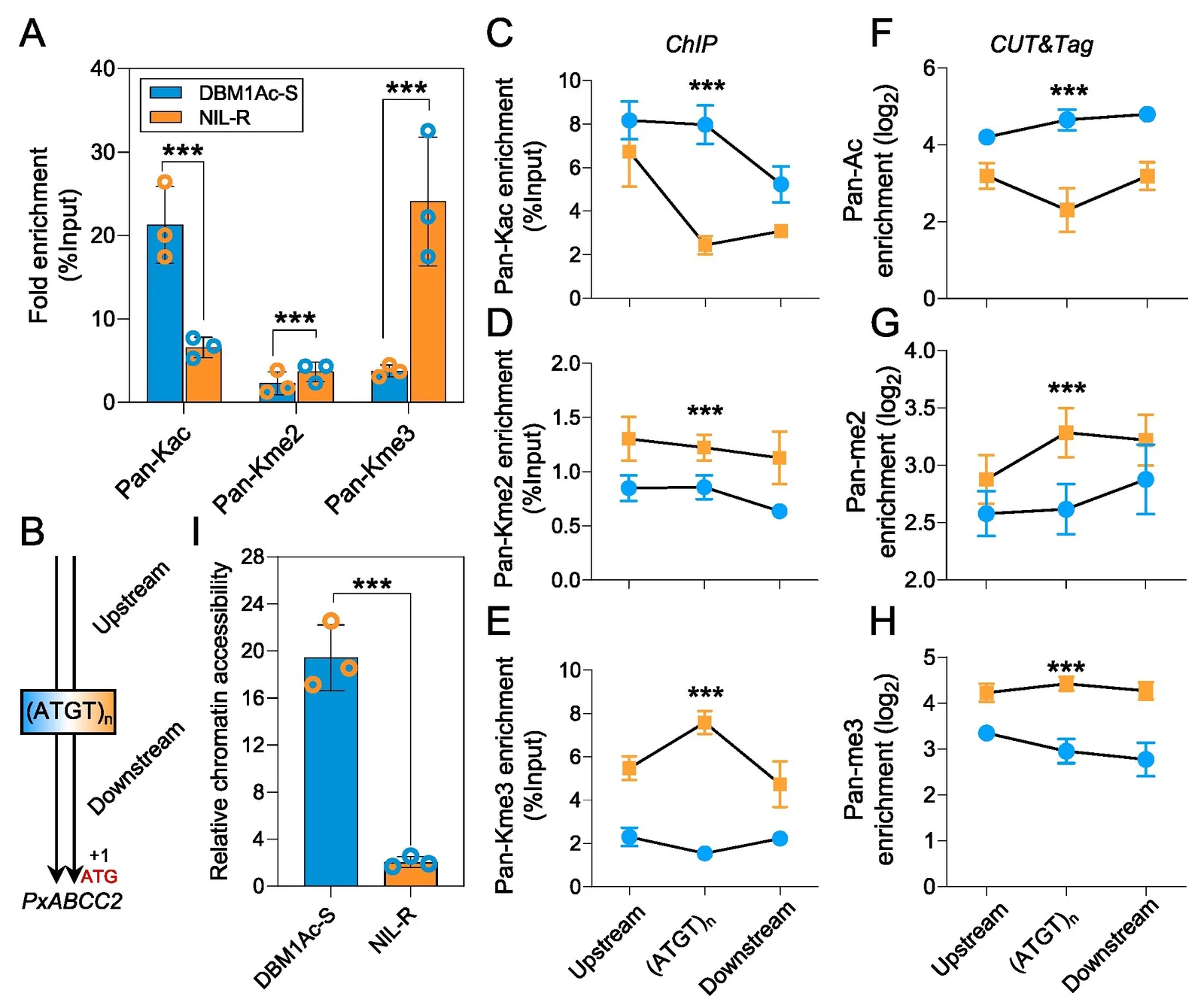
Microsatellite expansion induces differential histone modification and chromatin remodeling. (**A**) ChIP-qPCR quantification of histone methylation and acetylation binding to ATGT sites in the susceptible and resistant *ABCC2* promoter region. (**B**) Diagram of the region for histone modification detection. Levels of Pan-Kac (**C**), Pan-Kme2 (**D**), and Pan-Kme3 (**E**) at ATGT repeat region and its upstream and downstream 200–300 bp surrounding the *ABCC2* promoter locus assayed by ChIP-qPCR. Levels of Pan-Kac (**F**), Pan-Kme2 (**G**), and Pan-Kme3 (**H**) at ATGT repeats region and its upstream and downstream 200–300 bp surrounding the *ABCC2* promoter locus assayed by CUT&Tag-qPCR. CUT&Tag-qPCR results are presented as log_2_(spike-in-normalized enrichment). (**I**) qPCR analysis of chromatin accessibility of the ATGT repeat region in the DBM1Ac-S and NIL-R strains. Statistical significance was tested by one-way ANOVA with Tukey’s test. Data were presented as mean values ± SEM, *n* = 3, ns, not significant, ***P < .001.

To directly assess whether histone modification patterns across the *ABCC2* promoter vary with ATGT repeat expansion, we examined both the repeat-containing region and its flanking promoter sequences using two independent chromatin profiling approaches. ChIP-qPCR analyses revealed increased histone methylation and reduced histone acetylation in resistant strains carrying expanded ATGT repeats, whereas susceptible strains with short repeats exhibited the opposite pattern (Fig. 5B–E). Notably, these differences were most pronounced at the ATGT repeat region and gradually diminished in upstream and downstream promoter regions. To independently validate these findings, we performed CUT&Tag-qPCR targeting the same genomic intervals. CUT&Tag profiles recapitulated the ChIP-qPCR results (Fig. 5F–H). Consistent across both assays, histone modification patterns in the flanking promoter regions varied in concert with the presence and expansion of the ATGT microsatellite.

Consistent with these epigenetic changes, chromatin accessibility assays indicated that the *ABCC2* promoter adopts a more open configuration in susceptible strain but becomes relatively condensed in resistant strain harboring expanded repeats (Fig. 5I). Collectively, these data indicated that microsatellite expansion is closely associated with changes in histone modification patterns and chromatin structure at the *ABCC2* promoter, providing a mechanistic link between repeat length variation and transcriptional repression.

### Microsatellite expansion recruits E(z) to reshape chromatin and silence *ABCC2*

To further elucidate the mechanism by which the expanded ATGT repeats represses *ABCC2* expression, we focused on identifying and validating the histone modification enzymes responsible for chromatin remodeling at the ATGT repeat region. One of the nine proteins identified in both the (ATGT)_34_ Y1H and pull-down assays, but absent in the (ATGT)_5_ pull-down, was the E(z) methyltransferase. Despite the confirmed interactions with the ATGT repeat region, quantitative analysis revealed no significant changes in its mRNA level between resistant and susceptible strains, or after Bt toxin exposure (Fig. 6A). This suggested that the enrichment of E(z) in the association studies was not due to an increased expression of the protein. Notably, spatiotemporal expression profiling further showed that E(z) is highly expressed in the heads and eggs (Supplementary Fig. S5C), suggesting that this enzyme is broadly available *in vivo* and may be selectively co-opted at specific genomic loci rather than being resistance-inducible per se. To test whether it was due to selective recruitment by the repeat region, we performed ChIP-qPCR to examine specific histone marks around the microsatellite region. This revealed prominent methylation differences at H3K27 and H3K9 (Fig. 6B and Supplementary Fig. S10) and aligning with the increased methylation seen in the resistant insects. These findings align with the broader trend of increased methylation in resistant strains with expanded ATGT repeats. To directly assess the functional role of this enzyme, we silenced *PxE(z)* expression in third-instar larvae from the resistant strain or performed pharmacological inhibition using EED-targeting PRC2 inhibitor A-395. Both knockdown and inhibitor treatment resulted in decreased methylation at the ATGT repeat locus and significant upregulation of *ABCC2* mRNA (Fig. 6D–F). Importantly, larvae subjected to these treatments exhibited significantly increased mortality upon exposure to a median lethal concentration of Cry1Ac compared with control groups (Supplementary Fig. S11A), demonstrating that disruption of E(z)-mediated repression restores Bt susceptibility, providing evidence for the role of this enzyme in the epigenetic control of *ABCC2* expression.

**Figure 6.**
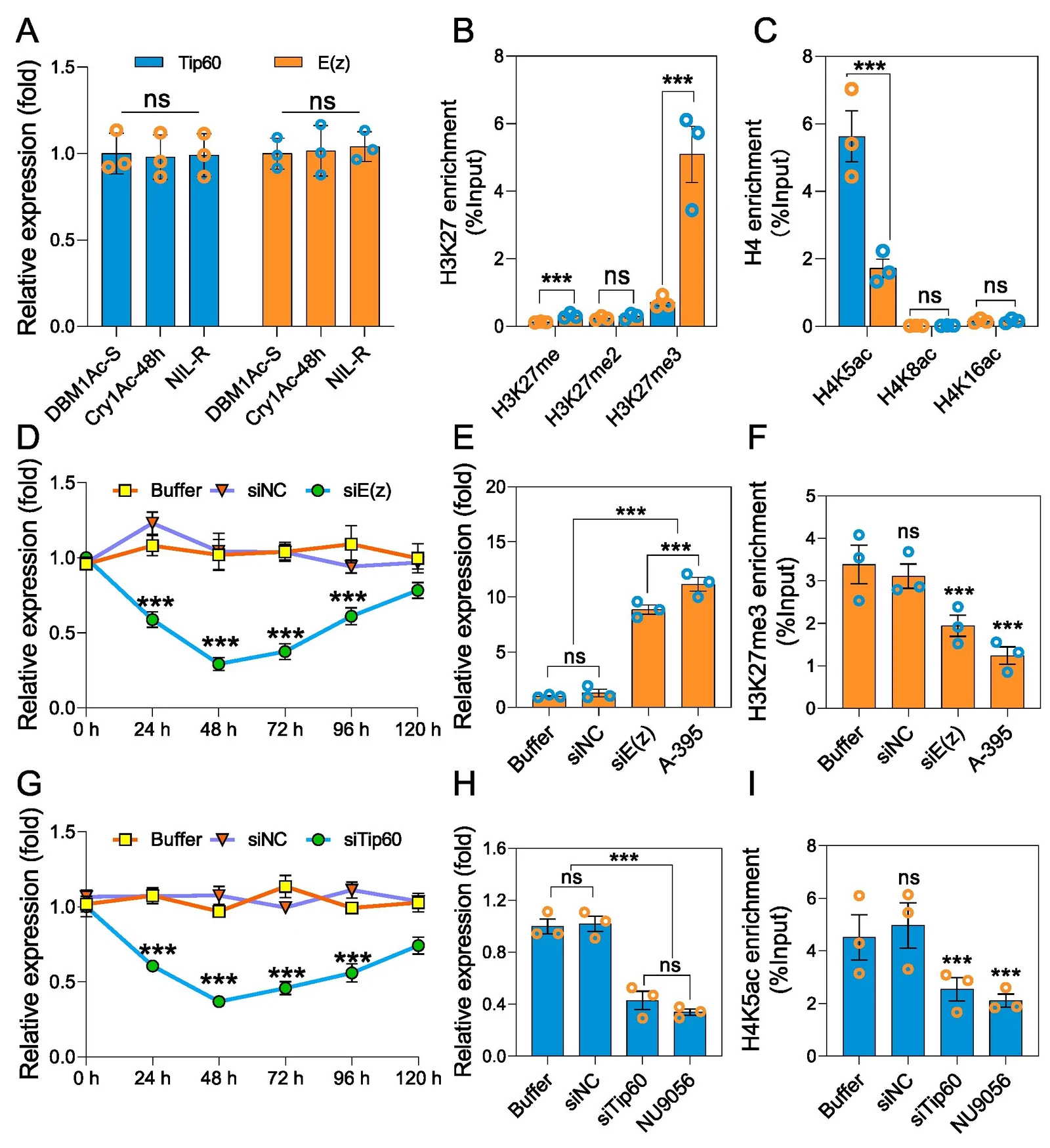
E(z) and Tip60 regulate the expression of *ABCC2* through H3K27me3 and H4K5ac, respectively. (**A**) Relative expression levels of the *PxE(z)* and *PxTip60* genes in the midgut of susceptible, Cry1Ac-treated, and resistant strains. The relative expression levels of the genes have been normalized to the expression level of the internal control gene *RPL32* and the expression levels in the DBM1Ac-S strain set to 1. (**B**) ChIP-qPCR quantification of H3K27me, H3K27me2, H3K27me3 and (**C**) H4K5ac, H4K8ac, and H4K16ac in the ATGT repeat region. (**D**) siRNA-mediated silencing of *PxE(z)* was detected at the transcriptional levels in the NIL-R strain. (**E**) Relative expression levels of the *PxABCC2* gene and (**F**) ChIP-qPCR quantification of H3K27me3 in the ATGT repeat region in the midgut of *siPxE(z)*-treated, A-395-treated, and NIL-R strains. The relative expression levels of the genes have been normalized to the expression level of the internal control gene *RPL32* and the expression levels in the NIL-R strain set to 1. (**G**) siRNA-mediated silencing of *PxTip60* was detected at the transcriptional levels in the DBM1Ac-S strain. (**H**) Relative expression levels of the *PxABCC2* gene and (**I**) ChIP-qPCR quantification of H4K5ac in the ATGT repeat region in the midgut of *siPxTip60*-treated, NU9056-treated, and DBM1Ac-S strains. The relative gene expression levels have been normalized to the expression level of the internal control gene *RPL32* and the expression levels in the DBM1Ac-S strain set to 1.Data of A-I are presented as mean values ± SEM, *n* = 3 biologically independent samples, ns, not significant, ***P < .001, one-way ANOVA with Tukey’s test was used for comparison.

### The potential role of histone acetylation on *ABCC2* expression

Examination of the (ATGT)_5_ pull-down data revealed the presence of the histone modifying acetyltransferase Tip60 (Fig. 4C and Supplementary Fig. S4), an enzyme that was not found in either the (ATGT)_34_ Y1H or pull-down assays. As with E(z), there was no difference in expression levels between susceptible and resistant insects (Fig. 6A); spatiotemporal expression profiling further showed that Tip60 is highly expressed in the testes and in adult males (Supplementary Fig. S5D). ChIP-qPCR revealed that acetylation differences were concentrated at H4K5 (Fig. 6C). Use of RNAi or the Tip60 inhibitor on the susceptible strain both led to decreased acetylation levels at the ATGT repeat region and downregulation of *ABCC2* expression (Fig. 6G–I). Consistent with the transcriptional effects, larvae subjected to Tip60 knockdown or inhibition displayed significantly reduced mortality following exposure to a diagnostic concentration of Cry1Ac relative to control groups (Supplementary Fig. S11E), indicating compromised susceptibility to Bt toxin. These results demonstrated that while Tip60 promotes acetylation and normal *ABCC2* expression in the susceptible strain, E(z) is recruited by the extended repeat sequence in the resistant strain, mediates methylation, and results in *ABCC2* gene silencing in the resistant strain. Consistent with their functional roles in chromatin regulation, immunofluorescence analysis revealed that both E(z) and Tip60 were predominantly localized in the nucleus and exhibited strong colocalization with their respective histone modification marks (Supplementary Fig. S12), further supporting their direct involvement in regulating chromatin states at the *ABCC2* locus. Additionally, to exclude effects of the injection procedure, DMSO vehicle, or inhibitor toxicity, we compared larvae treated with injection buffer, DMSO plus injection buffer, or the corresponding inhibitor in the absence of Cry1Ac. Neither vehicle affected *ABCC2* expression or histone modification levels, and neither inhibitor significantly affected larval survival (Supplementary Fig. S11), supporting the conclusion that the observed molecular and phenotypic effects were not attributable to the injection procedure, DMSO, or direct inhibitor toxicity.

### Microsatellite length and chromatin state partially revert under relaxed selection

To determine whether the microsatellite-chromatin relationship defined in the DBM1Ac-S/NIL-R background extends across strains and remains responsive to selection, we first compared repeat composition, *ABCC2* expression, and local histone marks among multiple susceptible and resistant strains. Across strains, short ATGT alleles were consistently associated with higher *ABCC2* expression, enrichment of the activating mark H4K5ac, and reduced H3K27me3, whereas expanded alleles showed the opposite pattern (Fig. 7A and B; Supplementary Fig. S13). Notably, the SZ-R strain, which carries relatively fewer repeats than other resistant strains (Fig. 7A), displayed a correspondingly intermediate molecular profile, supporting a quantitative association between repeat length and promoter state.

**Figure 7.**
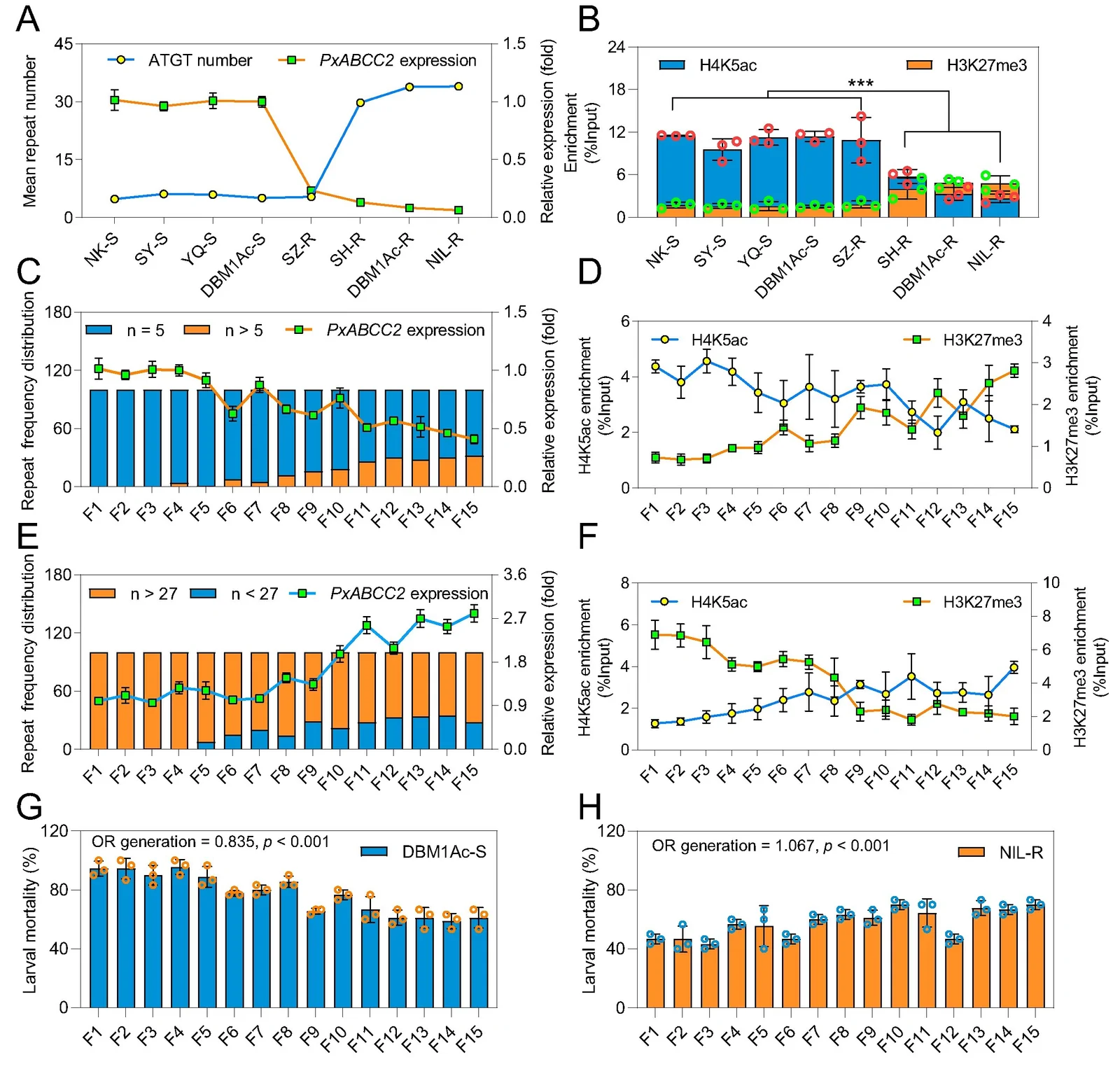
Microsatellite length, gene expression, local histone modifications, and Cry1Ac mortality under different selection regimes. (**A**) Mean ATGT repeat number at the *ABCC2* promoter and relative *PxABCC2* expression levels in multiple susceptible and resistant *P. xylostella* strains. (**B**) H4K5ac and H3K27me3 enrichment, expressed as percent input, at the repeat-containing region of the *ABCC2* promoter in the same strains. (**C, D**) Dynamics of repeat composition, *PxABCC2* expression, and local histone modifications in the susceptible DBM1Ac-S strain under continuous high-dose Cry1Ac selection across 15 generations. In (C), blue bars indicate individuals with five repeats, and orange bars indicate individuals with repeat numbers >5. In panel (D), relative *PxABCC2* transcript levels and percent-input enrichment of H4K5ac and H3K27me3 at the repeat-containing promoter region are shown across generations. (**E, F**) Dynamics of repeat composition, *PxABCC2* expression, and local histone modifications in the resistant NIL-R strain maintained in the absence of Bt selection across 15 generations. In panel (E), orange bars indicate individuals with repeat numbers >27, and blue bars indicate individuals with repeat numbers <27. In panel (F), relative *PxABCC2* transcript levels and percent-input enrichment of H4K5ac and H3K27me3 at the repeat-containing promoter region are shown across generations. (**G, H**) Longitudinal changes in larval mortality following Cry1Ac exposure across 15 generations of the DBM1Ac-S selection lineage (G) and the NIL-R relaxed-selection lineage (H). Bars represent the mean mortality of three independent bioassay groups, error bars indicate SEM, and individual points represent the biological replicates, each comprising 30 larvae. Mortality was analyzed from the numbers of dead and surviving larvae using a generalized linear model with a binomial error distribution and logit link, with biological replicate included as a blocking factor. Generation was modeled as a continuous predictor to evaluate the longitudinal trend within each population. Mortality decreased across generations in DBM1Ac-S (odds ratio per generation = 0.835, 95% CI = 0.806–0.864, *P* < .001) and increased across generations in NIL-R (odds ratio per generation = 1.067, 95% CI = 1.043–1.093, *P* < .001). Statistical significance was tested by one-way ANOVA with Tukey’s test. Data are presented as mean values ± SEM, *n* = 3, ns, not significant, *P < .05; **P < .01; ***P < .001.

We next tested whether this relationship could be dynamically reshaped under altered selection regimes. Under high-dose Cry1Ac selection, the susceptible strain exhibited a marked shift in repeat composition, with expanded alleles emerging from generation 6 onward. This shift was accompanied by reduced *ABCC2* expression, decreased H4K5ac, and increased H3K27me3, consistent with the establishment of a repressive chromatin state (Fig. 7C and D). Concordantly, bioassays performed at each generation revealed a progressive decrease in larval mortality upon Cry1Ac exposure, indicating the gradual acquisition of resistance (Fig. 7G). Conversely, when Bt selection was relaxed in the resistant strain, short-repeat individuals reappeared from generation 5 and persisted at low but fluctuating frequencies. This reversion was associated with increased *ABCC2* expression, elevated H4K5ac, and reduced H3K27me3 (Fig. 7E, F). In parallel, larval mortality progressively increased over generations, consistent with a partial restoration of Cry1Ac susceptibility (Fig. 7H). Together, we found that susceptible lineage partially acquire resistance under toxin selection, whereas resistant lineage partially revert under relaxed selection. These coordinated changes, considered together with the consistent associations observed across multiple susceptible and resistant strains, indicating that microsatellite length serves as a selection-responsive and reversible *cis*-regulatory module that dynamically rewires chromatin state to regulate gene expression and phenotypic resistance.

## Discussion

In this study, we identify microsatellite expansion as an epigenetic switch that confers insect resistance to Bt bioinsecticides (Fig. 8). Rather than relying on classical coding mutations or frameshift disruptions, this novel mechanism operates through repeat-length-dependent chromatin reprogramming at the *ABCC2* locus, leading to stable transcriptional silencing of this key toxin receptor in resistant *P. xylostella* strains. Specifically, expansion of a promoter TR shifts the chromatin landscape from an acetylation-associated active state to a methylation-dominated repressive state, thereby establishing a heritable and reversible “chromatin tuning knob.” These findings highlight microsatellite length as a *cis*-regulatory parameter capable of reconfiguring chromatin states and driving rapid adaptive responses to insecticide pressure.

**Figure 8.**
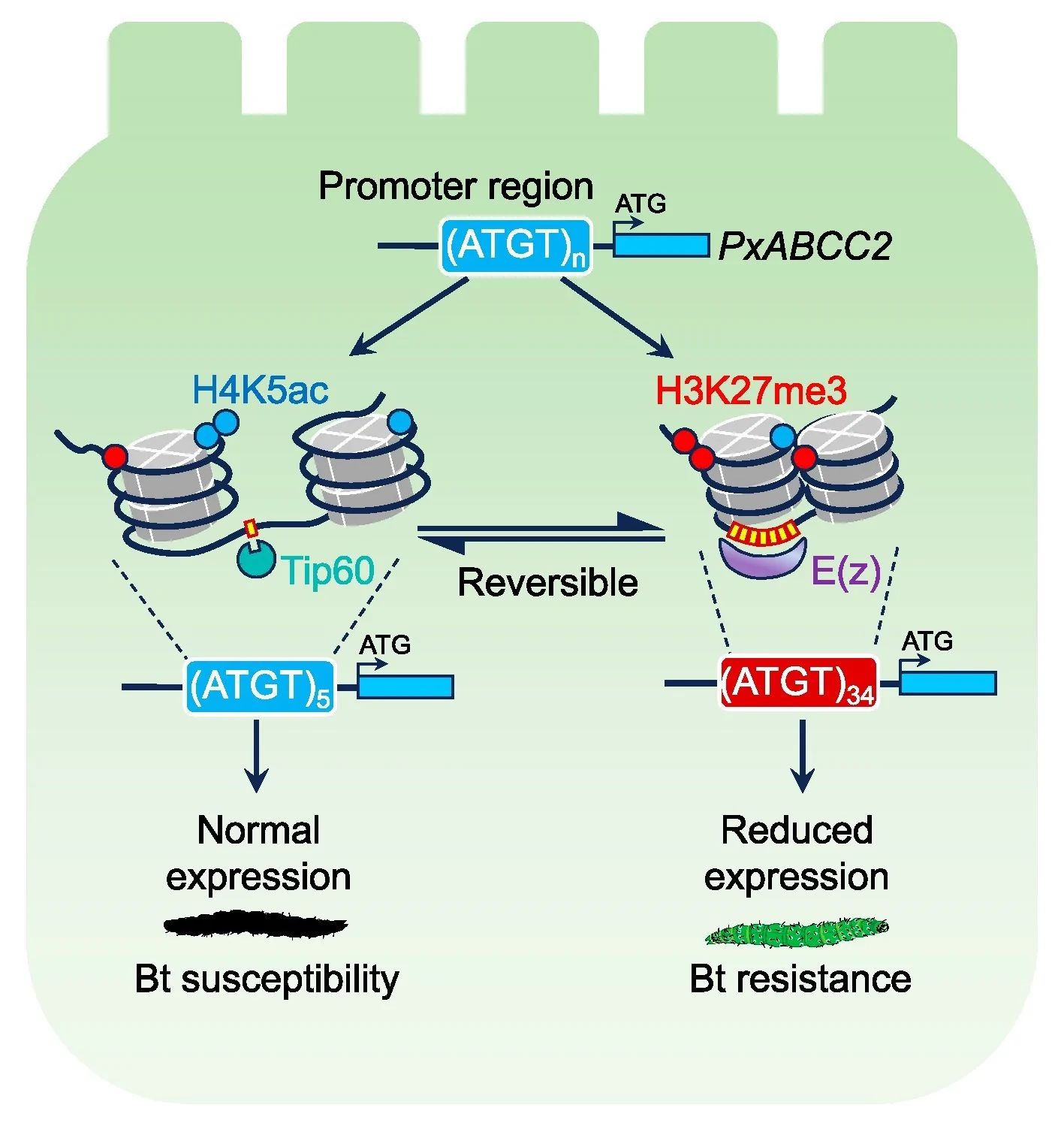
Proposed model linking ATGT microsatellite length to alternative chromatin states, *PxABCC2* expression, and Cry1Ac resistance. In the susceptible state, the short (ATGT)5 allele selectively associates with Tip60 *in vitro* and is linked to increased H4K5ac, an active chromatin configuration, and normal *PxABCC2* expression, thereby maintaining susceptibility to Cry1Ac. In the resistant state, the expanded (ATGT)34 allele selectively associates with E(z) *in vitro* and is proposed to facilitate the recruitment or stabilization of endogenous E(z)-containing PRC2 at the *PxABCC2* promoter. The resulting enrichment of H3K27me3 is associated with chromatin compaction and reduced *PxABCC2* expression, thereby contributing substantially to Cry1Ac resistance. The bidirectional arrow represents the coordinated shifts in repeat composition, chromatin state, and *PxABCC2* expression observed under altered selection regimes, rather than direct demonstration of rapid interconversion between the two states at a fixed allele.

Although *cis*-mutations disrupting the CDS of the *ABCC2* gene are a well-established cause of high-level Bt resistance in many lepidopteran species [10], accumulating evidence indicates that Bt resistance in lepidopteran insects can also be attributed to the downregulation of *ABCC2*, rather than coding mutations [12]. In *P. xylostella*, we previously demonstrated that a hormone-activated MAPK signaling cascade orchestrates the coordinated repression of midgut receptor genes, including *ABCC2*, through *trans*-acting regulatory networks [19–21, 23, 24, 26]. Here, we identify a fundamentally distinct regulatory layer in which expansion of a promoter microsatellite functions as a *cis*-encoded determinant of gene silencing. Unlike *trans*-regulatory pathways that rely on dynamic signaling inputs and TF activity, this mechanism is embedded in noncoding structural variation and operates through length-dependent chromatin remodeling at the promoter. Hence, these findings delineate a multilayered regulatory landscape in which *trans*-acting signaling pathways and *cis*-encoded chromatin mechanisms converge to enforce stable receptor repression. This integration expands the transcriptional regulatory repertoire governing *ABCC2* expression, coupling dynamic signaling inputs with structurally encoded chromatin states to achieve robust and persistent transcriptional silencing under strong selective pressure.

The causal contribution of repeat expansion is supported by the S-(ATGT)_34_ knock-in strain, which showed marked *ABCC2* repression and a 298-fold increase in Cry1Ac resistance. Nevertheless, because this strain was maintained collectively rather than as independently established knock-in lines, undetected founder-associated or background effects cannot be entirely excluded. The substantially higher resistance of NIL-R indicates that microsatellite expansion-induced ABCC2 repression accounts for a major but incomplete component of the phenotype. This difference is consistent with functional redundancy among Cry1Ac receptors: complete knockout of *PxABCC2* or *PxABCC3* confers 724-fold and 413-fold resistance, respectively, whereas simultaneous disruption of multiple receptors produces synergistically higher resistance. Thus, in a susceptible background, the expanded microsatellite establishes stable constitutive repression of *ABCC2* and a significant resistance phenotype, while additional resistance-associated changes result in the extreme resistance observed in NIL-R.

Microsatellites are highly mutable TRs that were long regarded as neutral genetic elements but are now increasingly recognized as functional components of the genome [52–54], Studies across diverse organisms have shown that their variations can influence gene expression, chromatin organization, and phenotypic traits [55–61]. However, mechanistic understanding of how microsatellite variations are translated into gene regulatory outcomes remains poorly explored. To our knowledge, microsatellite variation has not yet been functionally implicated in insect physiology. The only notable exception is a previous study showing that expansion of a promoter dinucleotide microsatellite (AC) enhances the expression of the cytochrome P450 gene *CYP6CY3*, thereby promoting the adaptation of the polyphagous aphid *Myzus persicae* to the tobacco alkaloid nicotine [62]. This elegant work provided important evidence that repeat-length variation can contribute to adaptive transcriptional regulation in response to xenobiotics in insects, while how variation at such repeat tracts generates distinct regulatory outcomes remains unclear. Our findings further extend this line of inquiry by showing that promoter microsatellite length can alter chromatin state to intricately govern gene expression. In this context, the ATGT repeat does not appear to act merely as a simple quantitative modulator of transcription, but rather as a length-sensitive regulatory element linked to differential engagement of opposing histone-modifying activities and stable gene repression. Thus, our study provides a new mechanistic framework for understanding how promoter repeat variation can be coupled to epigenetic regulation in insects, while also broadening current views of TRs as potentially evolvable components of regulatory architecture.

Strikingly, alleles carrying identical ATGT repeat motifs but differing in repeat length produce contrasting transcriptional outcomes, indicating that regulatory specificity cannot be simply explained by canonical motif-dependent protein–DNA recognition [63]. This observation challenges the conventional view that sequence identity alone determines binding specificity and instead points to repeat length as a critical regulatory parameter. Histone-modifying enzymes are typically recruited to chromatin through sequence-specific TFs within multiprotein complexes [64, 65]. However, our biochemical assays revealed that both Tip60 and E(z) exhibit repeat-length-dependent binding even in purified, cell-free conditions, suggesting that their selective association with the repeat tract does not strictly require auxiliary DNA-binding factors. These findings support an alternative mode of recruitment in which the intrinsic properties of repetitive DNA, which may be related to repeat length, local DNA conformation, or higher-order structural features, directly contribute to binding specificity. In this regard, the ATGT microsatellite functions not as a conventional motif-defined *cis*-regulatory element, but as a length-sensitive regulatory platform that selectively co-opts distinct chromatin-modifying activities and thereby reconfigures the local epigenetic atlas. An additional mechanistic question concerns the pronounced orientation dependence of the ATGT repeat. Although the forward and reverse constructs contain the same repeat length and nucleotide composition, reversing the tract largely abolished its repressive activity, suggesting that repeat orientation may affect DNA conformation, protein binding, or local chromatin organization. The structural basis of this direction-dependent regulation remains to be determined.

Of particular note, the same microsatellite locus is associated with distinct chromatin states in susceptible and resistant strains, marked by activating acetylation in one context and repressive methylation in the other. This resembles the competitive or cooperative interplay between histone marks reported, where H3K9 methylation and H3K27 acetylation converge to fine-tune transcription in a context-dependent manner [66]. This observation indicates that promoter repeat variation can bias the locus toward alternative epigenetic configurations with distinct regulatory outcomes. Because histone modifications are inherently dynamic [67, 68], the coupling between repeat length, chromatin state, and *ABCC2* expression may provide a regulatory architecture with considerable plasticity. A related unresolved question is why resistant alleles cluster around ~34 ATGT repeats rather than expanding further. Our reporter assays indicate that repression plateaus at ~32–35 repeats, suggesting limited additional regulatory or resistance benefits from longer tracts. Excessive expansion may instead increase replication slippage, genomic instability, aberrant transcription, or disruption of local chromatin organization, as observed in pathogenic human repeat-expansion disorders. Thus, the ~34-repeat state may reflect a balance between maximal *ABCC2* repression and the instability or fitness costs of further expansion, although this hypothesis requires direct measurements of repeat stability, mutation rates, and fitness across longer alleles. In this view, microsatellite variation could help stabilize repression under selection while still retaining the potential for flexible shifts in transcriptional state when regulatory or environmental contexts change. Notably, although microsatellites are widespread throughout the *P. xylostella* genome, repeat expansion appears to occur selectively rather than indiscriminately, raising the possibility that such variation is preferentially retained at loci where chromatin-based regulation confers adaptive value. In this case, microsatellite length variation may provide a flexible substrate upon which selection can act, linking genetic variability to epigenetic state transitions and, ultimately, to adaptive phenotypic outcomes. More broadly, these findings support a view of repetitive DNA as an active component of regulatory architecture rather than passive genomic “noise,” highlighting its potential role in shaping dynamic and context-dependent gene regulation.

Several limitations should be considered when interpreting this model. Although the Y1H, pull-down, EMSA, and competition assays support repeat-length- and sequence-selective binding *in vitro*, direct Tip60 and E(z) occupancy at the endogenous microsatellite loci could not be confirmed because the available antibodies were unsuitable for reliable ChIP. Thus, the locus-specific H4K5ac and H3K27me2/3 data indicate associated chromatin states rather than direct factor occupancy, and the *in vivo* H3K27 methylation is attributed to endogenous E(z)-containing PRC2. RNAi efficiency was confirmed at the transcript level, whereas the *in vivo* potency and specificity of A-395 and NU9056 in *P. xylostella* remain to be established. Nevertheless, the consistent effects of RNAi and inhibitor treatments on histone modifications, *ABCC2* expression, and Cry1Ac susceptibility support the proposed relationships, while future occupancy, protein-level, and rescue experiments would provide further validation.

Beyond its mechanistic implications, our findings further suggest that microsatellite-mediated chromatin regulation represents a general cross-kingdom strategy by which insects modulate gene expression in response to xenobiotic stress. Insects are continuously exposed to diverse synthetic and natural xenobiotics, including plant toxins, chemical and biological insecticides. The ability to tune expression of key receptor or detoxification genes through repeat-length variation and associated chromatin states provides a flexible regulatory mechanism for coping with these chemically diverse challenges. In this sense, microsatellite variation may define a broader regulatory framework underlying xenobiotic responses, extending beyond specific cases such as ABCC2-mediated Bt resistance described here and CYP6CY3-mediated nicotine tolerance reported previously. From an applied perspective, the strong coupling between promoter repeat length and transcriptional state highlights its potential as an excellent molecular marker for insecticide resistance surveillance. PCR-based detection of repeat length offers a rapid and cost-effective approach for monitoring field-evolved resistance, and more broadly, repeat-length polymorphisms may serve as versatile markers for tracking adaptive regulatory changes in insect populations exposed to diverse xenobiotic pressures.

## Supplementary Material

gkag1058_Supplemental_Files

## Data Availability

The full-length cDNA sequences of all the cloned genes in this study have been deposited in the GenBank database (Accession nos. PV959442-PV959452).
