# Supplementary material for: Microsatellite expansion redirects chromatin modifiers to confer insect resistance to Bt bioinsecticides
Source: Nucleic Acids Res. 2026 Oct 27;54(20):gkag1058. doi: 10.1093/nar/gkag1058 (PMC13710865; doi:10.1093/nar/gkag1058)
Supplement: Supplementary file 1 [file gkag1058_supplemental_files.zip › Supplementary Information.pdf]

Supplementary Information

Microsatellite expansion redirects chromatin modifiers to confer insect resistance to Bt bioinsecticides

Chunzheng Ouyang[1†], Kaiyue Sun[1†], Fu Wang[1], Fan Ye[1], Liuhong Zhu[1], Lina Dong[1], Yang Bai[1], Zhouqiang Cheng[1], Mingmei Wu[1], Neil Crickmore[2], Xuguo Zhou[3], Youjun Zhang[1], Zhaojiang Guo[1*]

[1] State Key Laboratory of Vegetable Biobreeding, Department of Plant Protection, Institute of Vegetables and Flowers, Chinese Academy of Agricultural Sciences, Beijing 100081, China

[2] School of Life Sciences, University of Sussex, Brighton BN1 9QE, UK

[3] Department of Entomology, School of Integrative Biology, College of Liberal Arts & Sciences, University of Illinois Urbana-Champaign, Illinois 61801-3795, USA

[*] To whom correspondence should be addressed. Tel: +86-10-82109518; Fax: +86-10-82109518; Email: guozhaojiang@caas.cn

This PDF file includes:

Figures S1 to S13

Tables S1 to S7

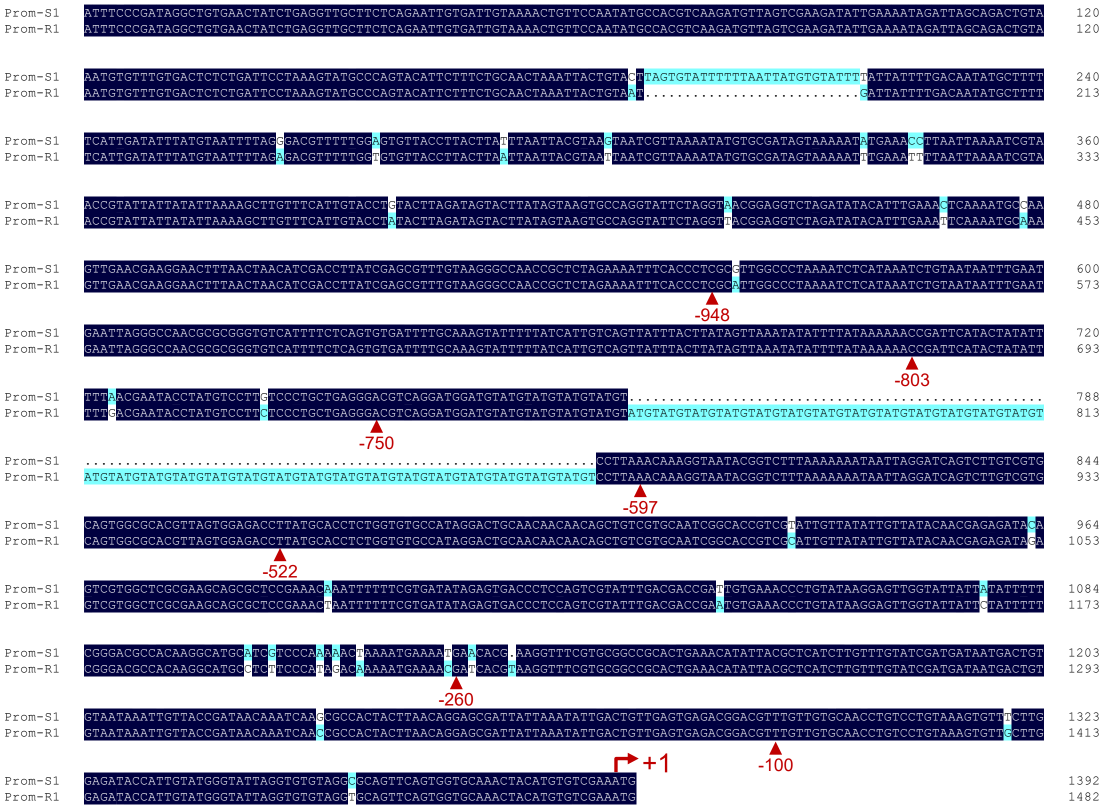

Supplementary Figure S1. Alignment of the two prominent promoter sequences of *ABCC2* cloned from susceptible DBM1Ac-S and resistant NIL-R strains. The translation starts site (ATG) indicated by +1 with a right arrow. The specific sites of the 5′ to 3′ deletions shown in Figure 1 are marked with red triangles.

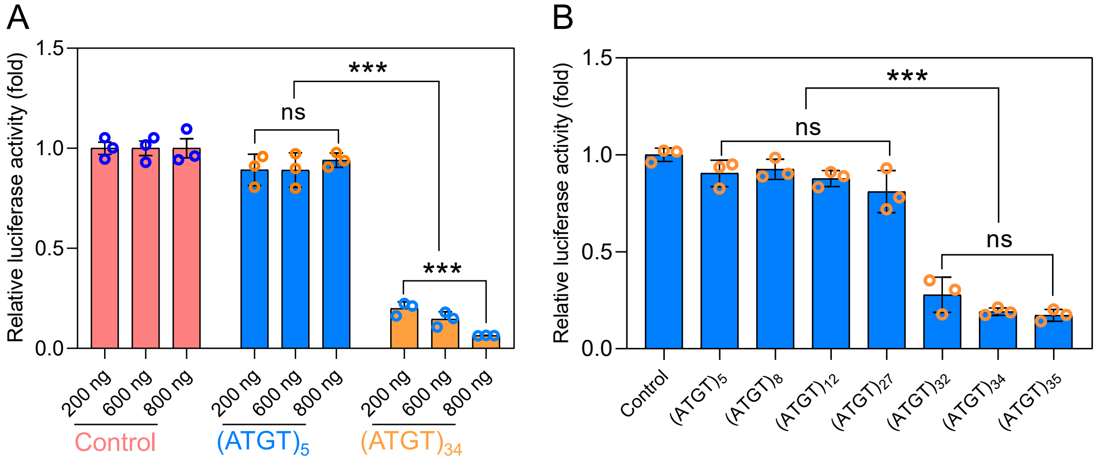

Supplementary Figure S2. Transcriptional activity of the microsatellites in different cell types. (**A**) Dose-dependent effects of copy number on transcriptional activity in S2 cells. (**B**) Transcriptional activity of different $(ATGT)_n$ copy numbers in 293T cells. Various numbers of ATGT repeats were inserted upstream of the minimal promoter in the pGL4.23 firefly luciferase reporter vector. These constructs were co-transfected

with the pGL4.73 Renilla luciferase plasmid driven by the SV40 early promoter into S2 and 293T cells. Relative luciferase activity was calculated as the ratio of firefly to Renilla luciferase signals and normalized against the activity of the empty pGL4.23 vector. Data represent means ± SEM from three independent biological replicates (n = 3). Statistical significance was determined by one-way ANOVA followed by Tukey's post hoc test: ns, not significant, ***$P < 0.001$.

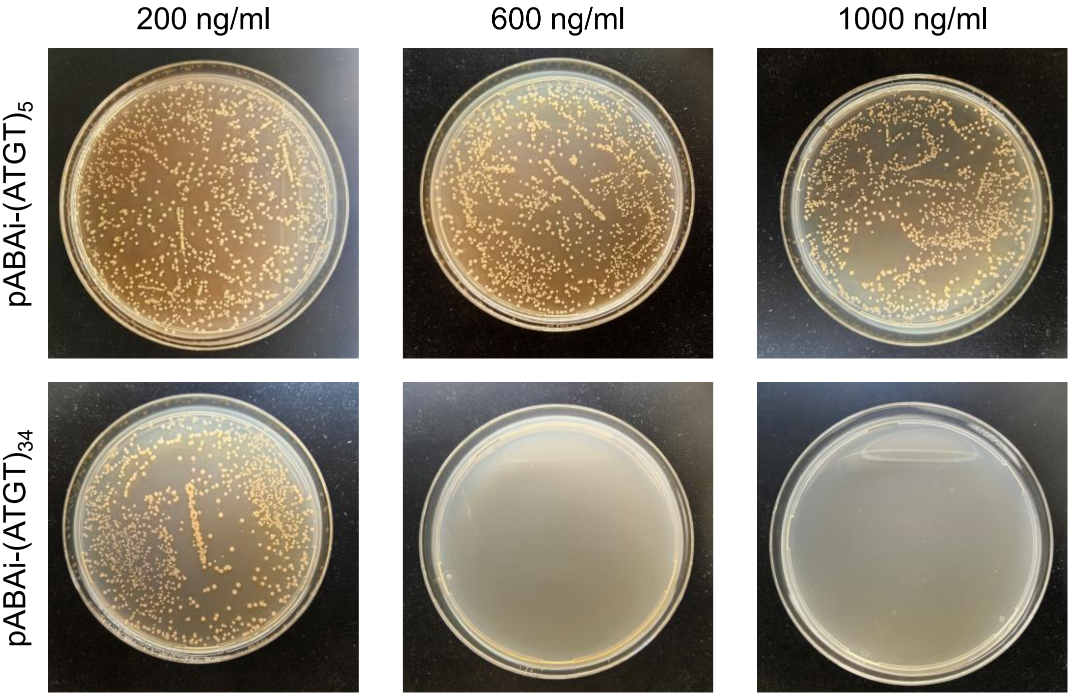

Supplementary Figure S3. Autoactivation assay of $(ATGT)_n$ in yeast. $(ATGT)_{5/34}$ sequences were integrated into the genome of the Y1H Gold yeast strain to generate different bait strains. Yeast cells were adjusted to an OD_{600} of 0.002, and 90 µL of each suspension was evenly spread onto SD/-Ura medium plates supplemented with varying concentrations of aureobasidin A (AbA). Growth patterns under different AbA concentrations are shown in the figure.

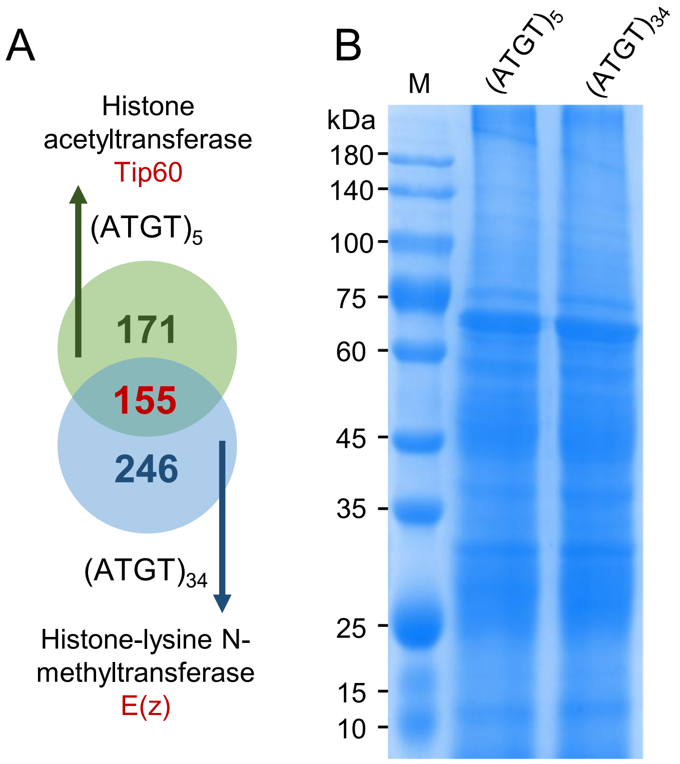

Supplementary Figure S4. Identification of $(ATGT)_n$-interacting proteins by pull-down assay. To identify proteins that specifically bind to the $(ATGT)_n$, a biotin-labeled $TR_{(ATGT)5/34}$ probe was synthesized and used to pull down midgut proteins extracted from susceptible and resistant *P. xylostella* strains. (**A**) A schematic diagram showing the capture of proteins by different $(ATGT)_n$ probes, with Tip60 [exclusive to $(ATGT)_5$] and E(z) [exclusive to $(ATGT)_{34}$] highlighted. (**B**) The proteins were separated by SDS-PAGE and visualized by Coomassie Brilliant Blue staining. Lane 1: protein molecular weight markers (kDa); Lane 2: proteins from the susceptible strain; Lane 3: proteins from the resistant strain.

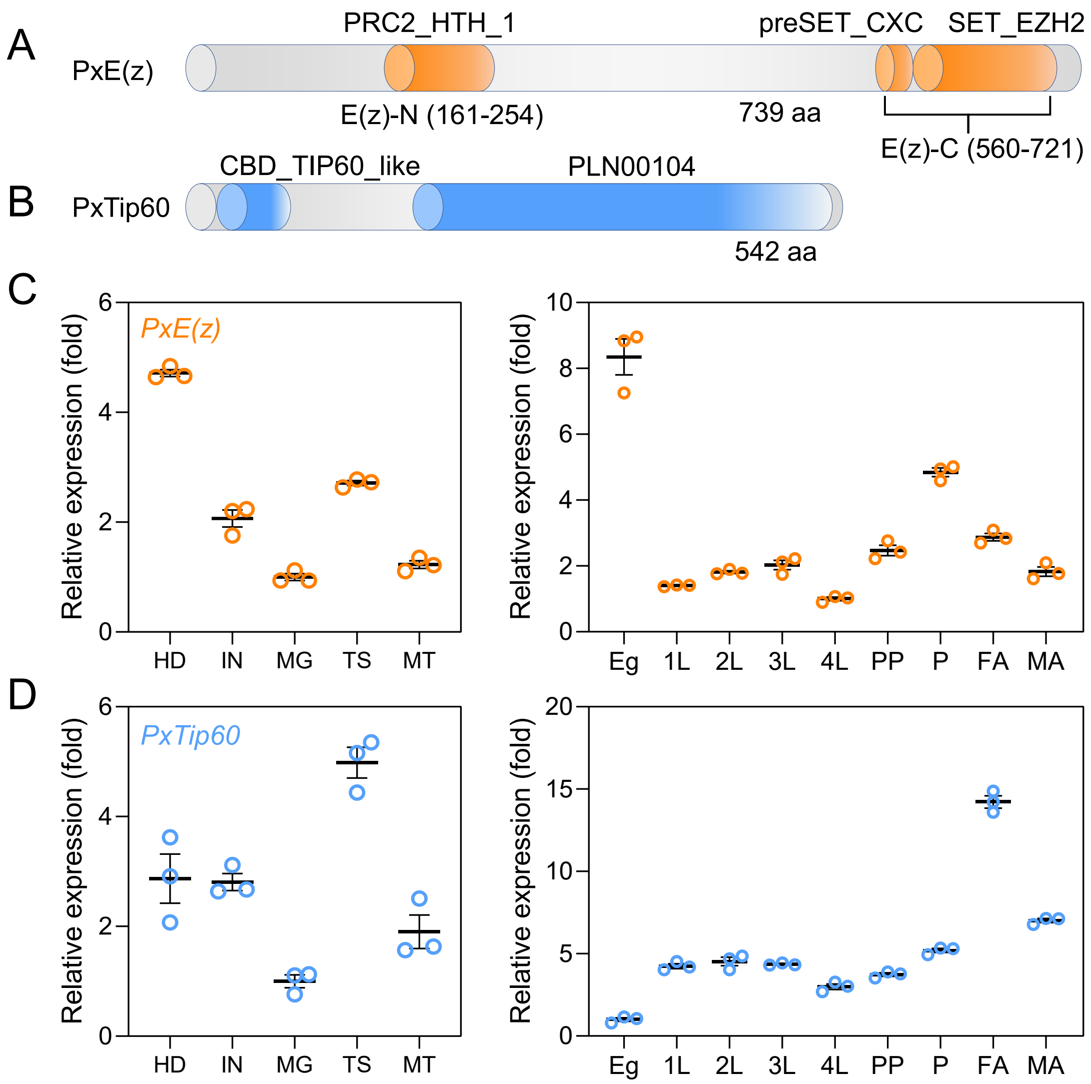

Supplementary Figure S5. Structural prediction and spatio-temporal expression analysis of *PxTip60* and *PxE(z)*. (**A**) Schematic diagram of the E(z) protein structures. (**B**) Schematic diagram of the Tip60 protein structures. (**C**) The relative expression of *PxE(z)* in different tissues and developmental stages. (**D**) The relative expression of *PxTip60* in different tissues and developmental stages. For different tissues (HD, head; IN, integument; MG, midgut; TS, testis; MT, Malpighian tubules) and developmental stages (EG, eggs; L1-4, first- to fourth-instar larvae; PP, prepupae; P, pupae; MA and FA, male and female adults).

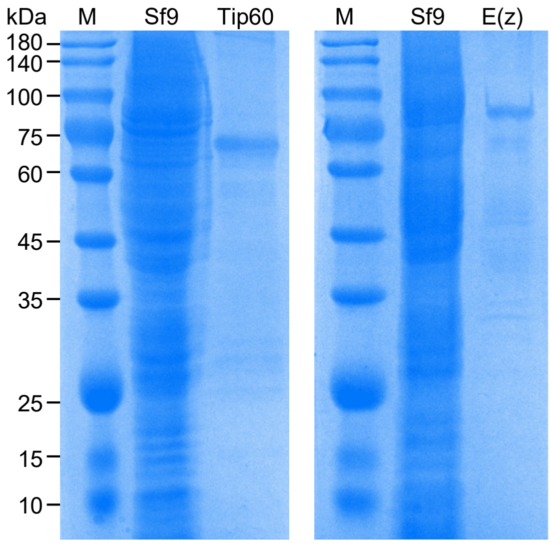

Supplementary Figure S6. Heterologous expression of the recombinant Tip60 and E(z) protein in Sf9 cell as detected by 10% SDS-PAGE. M: protein molecular weight markers (kDa); Sf9: supernatant of Sf9 cells disrupted by ultrasound; Tip60/E(z): purified recombinant Tip60 or E(z) protein.

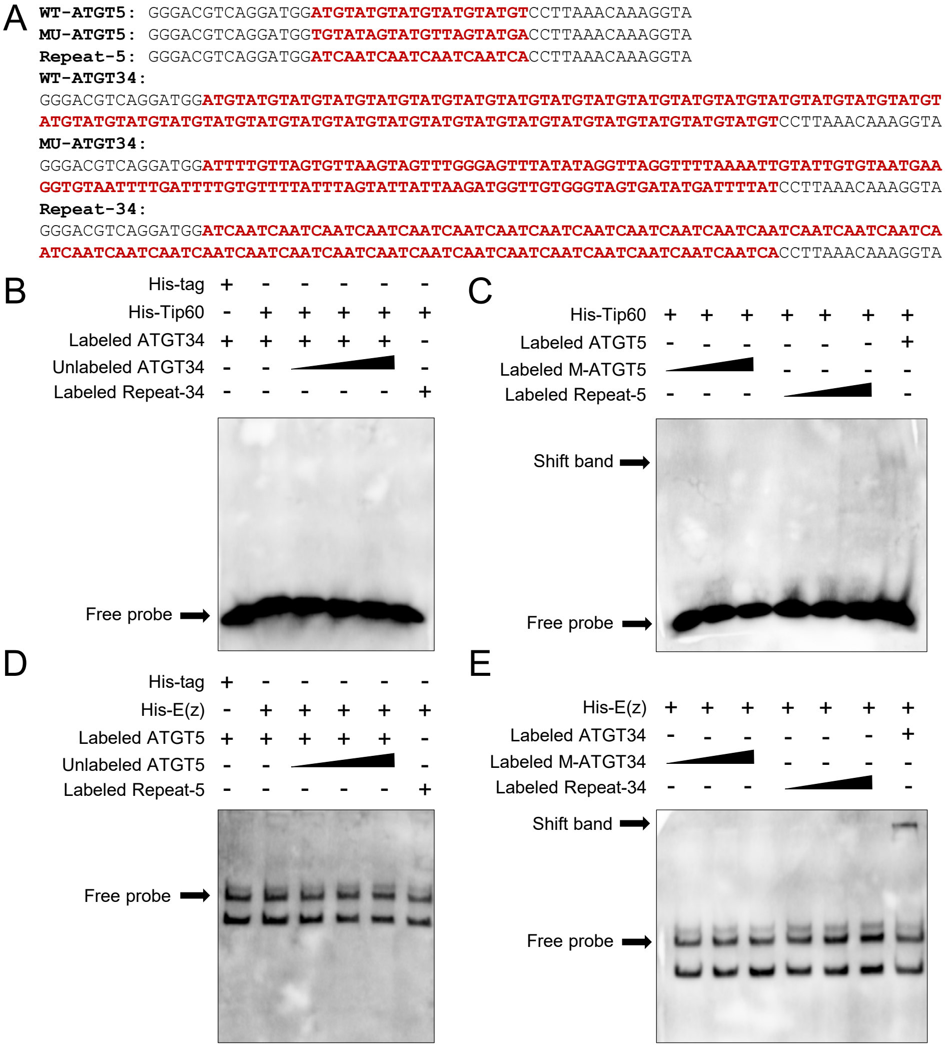

Supplementary Figure S7. EMSA validation of repeat-length- and sequence-specific binding of Tip60 and E(z) to the $(ATGT)_n$ microsatellite. (**A**) Sequences of the probes used in the EMSAs. (**B**) EMSA using the expanded-repeat $(ATGT)_{34}$ probe and purified Tip60. No detectable protein-DNA complex was observed, indicating that Tip60 did not bind the expanded-repeat probe under the tested conditions. (**C**) EMSAs using purified Tip60 and either a length-matched scrambled probe or a length-matched probe containing a different short tandem-repeat sequence. Neither control probe produced a detectable mobility shift. (**D**) EMSA using the short-repeat $(ATGT)_5$ probe and purified E(z). No detectable protein-DNA complex was observed, indicating that E(z) did not bind the short-repeat probe under the tested conditions. (**E**) EMSAs using purified E(z) and either a length-matched scrambled probe or a length-matched probe containing a different expanded tandem-repeat sequence.

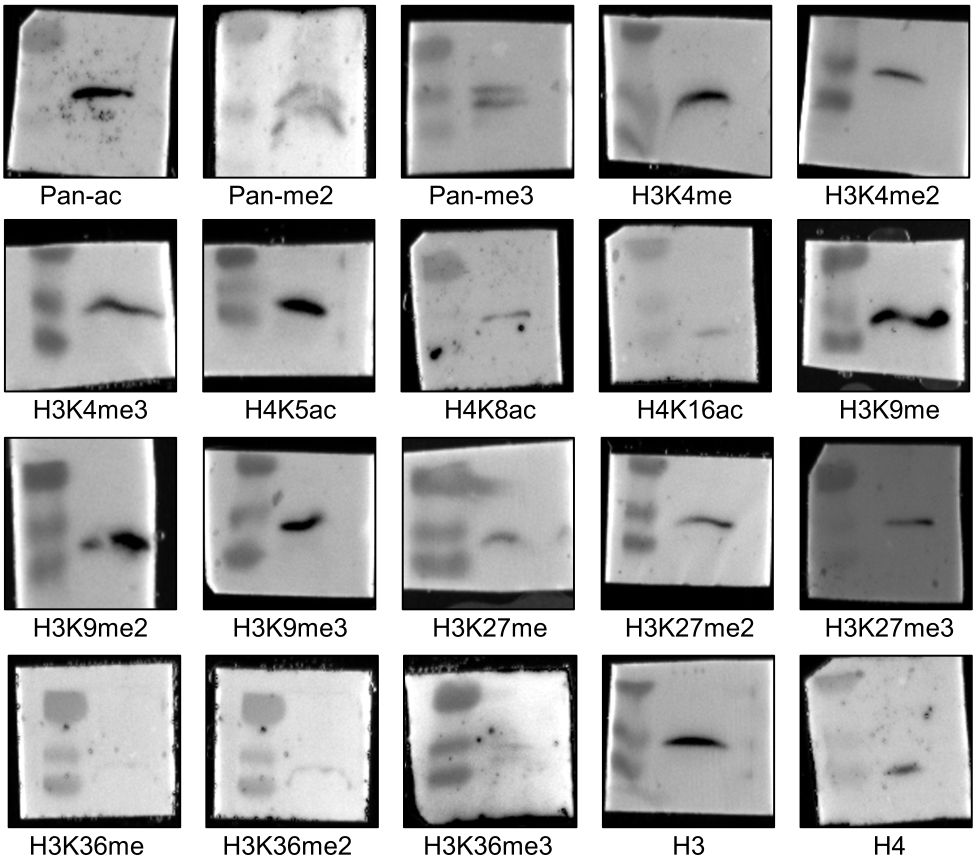

Supplementary Figure S8. Validation of histone modification antibodies by western blotting. Representative western blots showing the specificity of the antibodies against the indicated histone modifications and core histones. For each blot, the

molecular weight marker bands shown on the left correspond to 25, 15, and 10 kDa from top to bottom, respectively.

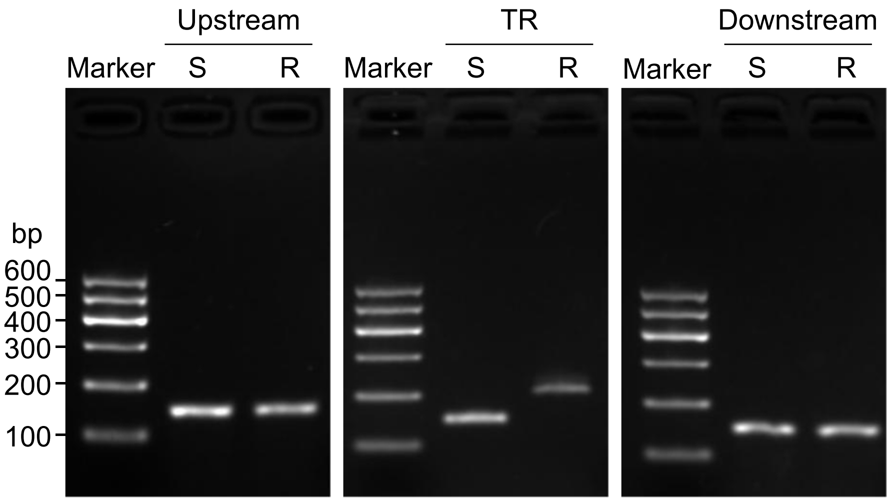

Supplementary Figure S9. Agarose gel electrophoresis validation of ChIP-qPCR amplicons. PCR products from the upstream, ATGT repeat-containing (TR), and downstream regions of the *ABCC2* promoter in susceptible DBM1Ac-S (S) and resistant NIL-R (R) strains. Expected amplicon sizes were 162 bp (Upstream), 120 bp (S) and 236 bp (R) (TR), and 162 bp (Downstream).

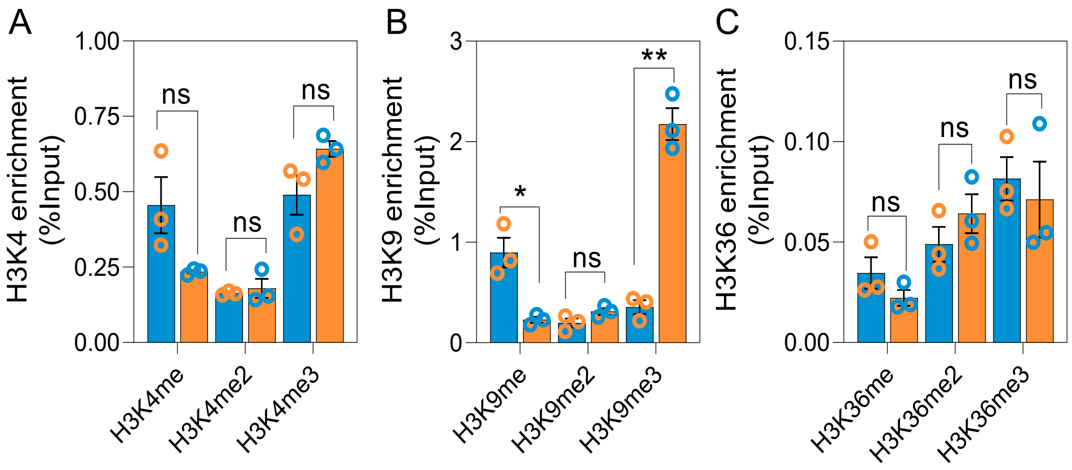

Supplementary Figure S10. Detection of site-specific histone modifications. ChIP-qPCR quantification of (**A**) H3K4me, H3K4me2, H3K4me3 and (**B**) H3K9me, H3K9me2, H3K9me3 and (**C**) H3K36me, H3K36me2, H3K36me3 in the $(ATGT)_{5/34}$ promoter region. Data are represented by the mean values ± SEM, n = 3 (A, B and C) biologically independent samples, statistical significance was calculated with Tukey's test: ns, not significant, *$P < 0.05$; **$P < 0.01$.

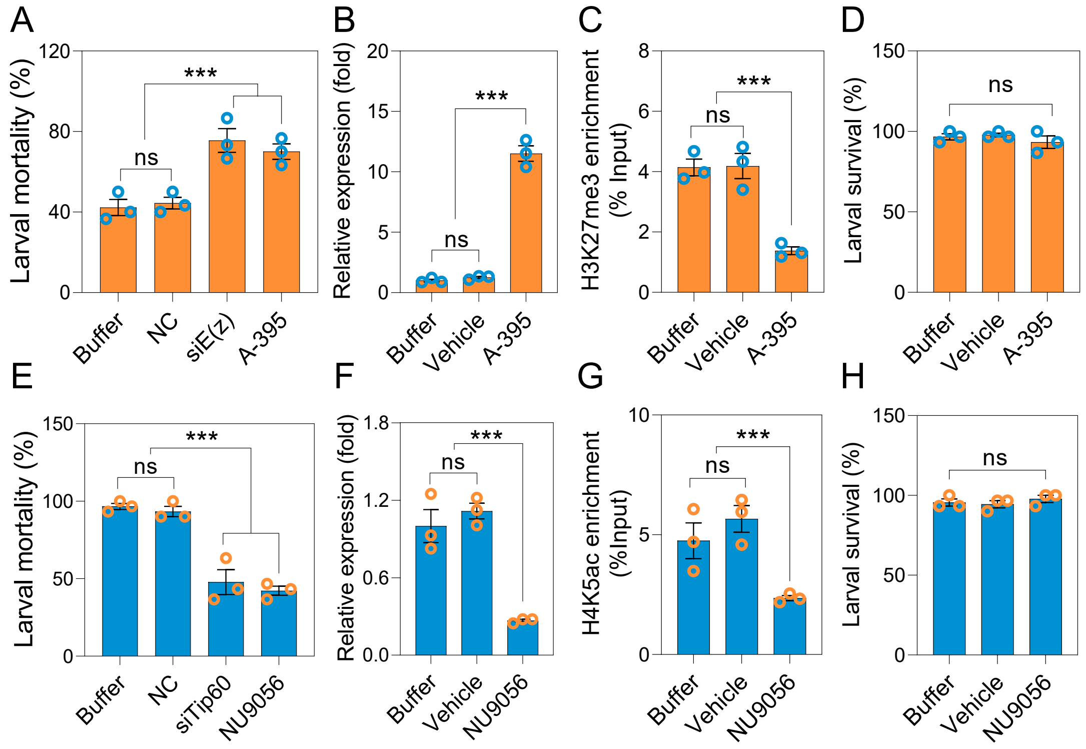

Supplementary Figure S11. Effects of E(z) and Tip60 perturbation on *PxABCC2* expression, histone modifications, and susceptibility to Bt Cry1Ac. (**A**) Effects of *PxE(z)* knockdown and pharmacological inhibition with A-395 on Cry1Ac-induced larval mortality in the resistant NIL-R strain. (**B-D**) Effects of A-395 treatment in NIL-R on (B) relative *PxABCC2* transcript abundance determined by RT-qPCR, (C) H3K27me3 enrichment at the $(ATGT)_n$-containing promoter region determined by ChIP-qPCR, and (D) larval survival at 48 h after injection. (**E**) Effects of *PxTip60* knockdown and pharmacological inhibition with NU9056 on Cry1Ac-induced larval mortality in the susceptible DBM1Ac-S strain. (**F-H**) Effects of NU9056 treatment in DBM1Ac-S on (F) relative *PxABCC2* transcript abundance determined by RT-qPCR, (G) H4K5ac enrichment at the $(ATGT)_n$-containing promoter region determined by ChIP-qPCR, and (H) larval survival at 48 h after injection. Buffer denotes the injection control, NC denotes the negative-control siRNA treatment, and vehicle denotes the DMSO-containing vehicle control with the same final DMSO concentration as the corresponding inhibitor treatment. Molecular data are presented as means ± SEM from three independent biological replicates, each calculated from the mean of four technical measurements. Cry1Ac bioassays and injection-survival assays were each performed with three independent groups of 30 larvae per treatment (90 larvae in

total). Molecular data were analyzed by one-way ANOVA followed by Tukey's multiple-comparison test. Mortality and survival data were analyzed using generalized linear models with a binomial error distribution and logit link, with pairwise comparisons adjusted using the Holm method. ns, not significant; ***P < 0.001.

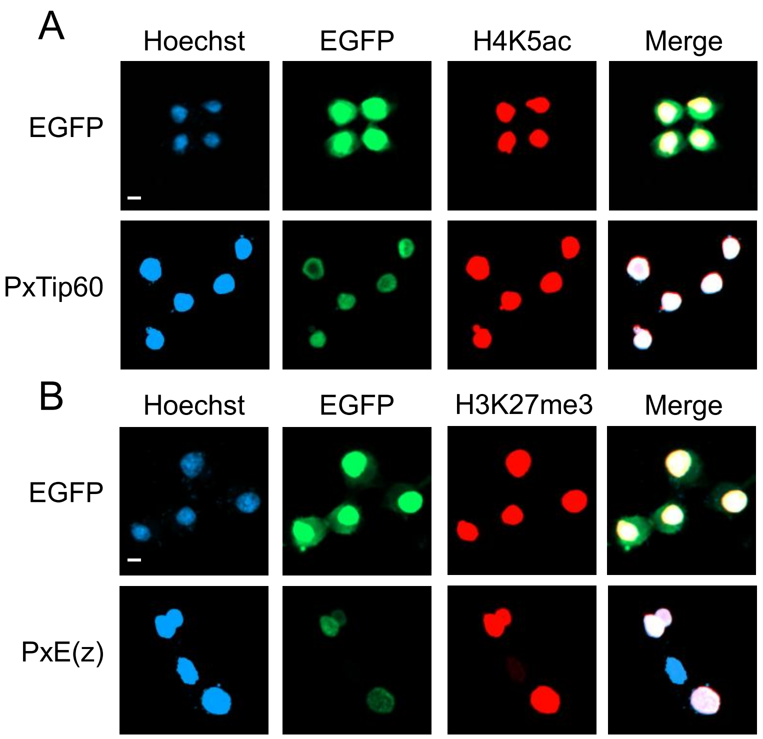

Supplementary Figure S12. Nuclear localization and colocalization of Tip60 and E(z) with their respective histone modifications in Sf9 cells. (**A**) Subcellular localization of Tip60 in Sf9 cells. (**B**) Subcellular localization of E(z) in Sf9 cells. Cells expressing EGFP alone or EGFP-tagged Tip60/E(z) were stained with Hoechst to label nuclei and immunostained for H4K5ac/H3K27me3. Merged images show nuclear localization of Tip60/E(z) and its colocalization with H4K5ac/H3K27me3 signals (Scale bar, 5 µm).

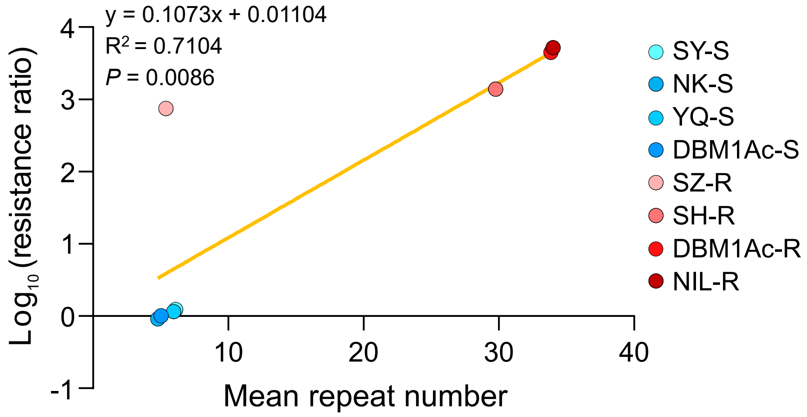

Supplementary Figure S13. Linear regression analysis between mean ATGT repeat number at the *ABCC2* promoter and $\log_{10}$-transformed resistance ratio to Bt Cry1Ac in different *P. xylostella* strains. DBM1Ac-R and NIL-R are field-evolved Cry1Ac-resistant strains. SZ-R is a laboratory-selected Cry1Ac-resistant strain, whereas SH-R is a laboratory-selected Btk-resistant strain. DBM1Ac-S, YQ-S, SY-S, and NK-S were collected from the field and are susceptible to Cry1Ac.

Supplementary Table S1. Susceptibility to Bt Cry1Ac toxin or Btk formulation of nine *P. xylostella* strains

Strains	Treatments	N*	LC_{50} (95% CL)†	Slope ± SE	χ^2(df)‡	RR§
DBM1Ac-S	Cry1Ac	210	0.71 (0.46-0.99)	1.97 ± 0.23	2.03(5)	1.00
	Btk	210	0.74 (0.57-0.96)	1.94 ± 0.23	4.71(5)	1.00
NK-S#	Cry1Ac	210	0.88 (0.69-1.12)	2.14 ± 0.24	3.58(5)	1.23
SY-S#	Cry1Ac	210	0.82 (0.64-1.05)	2.07 ± 0.24	3.81(5)	1.15
YQ-S#	Cry1Ac	210	0.65 (0.51-0.83)	2.11 ± 0.25	1.06(5)	0.91
DBM1Ac-R	Cry1Ac	210	3196.04 (2201.75-4947.69)	2.28 ± 0.28	7.34(5)	4501.46
NIL-R	Cry1Ac	210	3684.73 (2590.20-5612.34)	2.35 ± 0.29	6.73(5)	5189.76
SZ-R	Cry1Ac	210	527.45 (410.25-678.90)	1.95 ± 0.22	2.62(5)	742.89
SH-R	Btk	210	1015.95 (807.79-1290.01)	2.27 ± 0.26	4.17(5)	1372.91
S-$(ATGT)_{34}$	Cry1Ac	210	211.81 (159.94-294.25)	1.71 ± 0.21	4.78(5)	298.33

*Number of larvae tested (larvae of the control group not included).

†Concentration of Cry1Ac toxin (mg/L) killing 50% of larvae and its 95% confidence limits (CL).

‡The value of chi-square and degrees of freedom (df) were calculated by Polo Plus 2.0.

§RR: Resistance ratio (RR) calculated by LC_{50} of tested strains divided by LC_{50} of DBM1Ac-S.

#NK-S, SY-S and YQ-S are Bt Cry1Ac-susceptible strains collected from Beijing, China.

Supplementary Table S2. Likelihood-ratio tests of parallelism and equality of concentration–mortality regressions for different *P. xylostella* strains.

Treatment	Comparison	Parallelism LR χ^2	df	*P* value	Equality LR χ^2	df	*P* value
Cry1Ac	Overall comparison	4.71	7	0.695	1008.741	7	<0.0001
Cry1Ac	NK-S vs DBM1Ac-S	0.246	1	0.62	1.41	1	0.235
Cry1Ac	SY-S vs DBM1Ac-S	0.089	1	0.765	0.647	1	0.421
Cry1Ac	YQ-S vs DBM1Ac-S	0.166	1	0.683	0.262	1	0.609
Cry1Ac	S-(ATGT)$_{34}$ vs DBM1Ac-S	0.684	1	0.408	223.868	1	<0.0001
Cry1Ac	DBM1Ac-R vs DBM1Ac-S	0.728	1	0.394	259.78	1	<0.0001
Cry1Ac	SZ-R vs DBM1Ac-S	0.006	1	0.937	229.891	1	<0.0001
Cry1Ac	NIL-R vs DBM1Ac-S	1.02	1	0.312	264.657	1	<0.0001
Btk	SH-R vs DBM1Ac-S	0.922	1	0.337	247.433	1	<0.0001

Concentration–mortality data were analyzed using binomial probit regression based on the numbers of dead and surviving larvae at each concentration. The parallelism test compared a model containing strain-specific slopes and intercepts with a reduced model containing strain-specific intercepts and a common slope. Because the hypothesis of parallelism was not rejected, equality of the regression lines was subsequently evaluated by comparing the common-slope model containing strain-specific intercepts with a reduced model containing a common intercept and slope. Models were compared using likelihood-ratio χ^2 tests. Cry1Ac and Btk bioassays were analyzed separately. LR, likelihood ratio; df, degrees of freedom.

Supplementary Table S3. CRISPR/Cas9-induced $(ATGT)_{34}$ insertion in the DBM1Ac-S strain

Original strain	G_0				G_1			G_2	Mutant strain
	Eggs[#]	Hatched (%)	Adults (%)	Sib-cross[*]	Pupae	Genotypes (%)[‡]	Sib-cross[†]	Homozygotes (%)[¶]	
DBM1Ac-S	543	226/543 (41)	125/226 (55)	50♂× 50♀	118	27/118 (23)	—	—	S-$(ATGT)_{34}$
						34/118 (29)	—	—	
						43/118 (36)	—	—	
						14/118 (12)	6♂ × 8♀	22	

[#]A total of 543 fresh preblastodermal eggs from the DBM1Ac-S strain were collected for microinjection.

[*]50 male and 50 female moths from S-$(ATGT)_{34}$ groups were randomly selected to sibling crossed to produce G_1 progeny.

[‡]A total of 118 of G_1 were obtained for genotyping, four genotypes were included: 23% (27/118), WT; 29% (34/118), CRISPR/Cas9-induced NHEJ; 36% (43/118), heterozygous for $(ATGT)_n$ mutation; 12% (14/118), homozygous for $(ATGT)_{34}$, in the promoter of *ABCC2* in the DBM1Ac-S strain.

[†]Six male and eight female homozygotes were sib-mated to generate G_2 progeny.

[¶]22 G_2 individuals were homozygous for $(ATGT)_{34}$ insertion by sequencing.

Supplementary Table S4. Primer efficiency and amplicon analysis

Primer region	DNA template	Slope	Amplification efficiency (%)	R^2	Amplicon size (bp)
$(ATGT)_n$	DBM1Ac-S	−3.439	95.34	0.9987	120
	NIL-R	−3.485	93.62	0.9891	236
Upstream	DBM1Ac-S	−3.474	94.01	0.992	162
Downstream	DBM1Ac-S	−3.321	100.06	0.999	162

Supplementary Table S5. Primers used in this study

Purpose	Origin	Primer name	Primer sequence (5′-3′)
Promoter cloning	ABCC2	ABCC2-F	ATTTCCCGATAGGCTGTGAACTATC
		ABCC2-R	TTCGACACATGTAGTTTGCACCACT
pGL4.10 recombinant[†]	Susceptible promoter	S-P(-831/-1)-F	CGCGTTGGCCCTAAAATCTC
		S-P(-686/-1)-F	CCGATTCATACTATATTT
		S-P(-618/-1)-F	ACGTCAGGATGGATGTATGTATG
		S-P(-596/-1)-F	AACAAAGGTAATACGGTCTTTAAAA
		S-P(-521/-1)-F	TTATGCACCTCTGGTGTGCCA
		S-P(-260/-1)-F	TGAACACGAAGGTTTCGTGCG
		S-P(-100/-1)-F	TTGTTGTGCAACCTGTCC
	Resistant promoter	R-P(-948/-1)-F	CGCATTGGCCCTAAAATCTC
		R-P(-803/-1)-F	CCGATTCATACTATATTT
		R-P(-750/-1)-F	ACGTCAGGATGGATGTATGTATG
		R-P(-597/-1)-F	AACAAAGGTAATACGGTCTTTAAAA
		R-P(-522/-1)-F	TTATGCACCTCTGGTGTGCCA
		R-P(-260/-1)-F	GATCACGTAAGGTTTCGTGCGG
		R-P(-100/-1)-F	TTGTTGTGCAACCTGTCC
Gene cloning	SD	SD-F	AAGTGACAAATGGAAAAGTT
		SD-R	TGAATGTGCGATGTAAAGA
	CEBP	CEBP-F	ATGGAGTCCCCACAGATG
		CEBP-R	TCAGAGCGCGTGGTCGCC
	CAUP	CAUP-F	TGTTTAAGTGAAGCCGCCCA
		CAUP-R	CGCCCCACGTATCACTTTCT
	HOXA3	HOXA3-F	GACGTCCAGGTGAGGAAACC
		HOXA3-R	AAGTAATTAATAGTGAACCAGTTCG
	EXD	EXD-F	CGTGAATAGTGTTTACGGACGG
		EXD-R	TTTTTGCTTAACTGCCGCCC
	POU6F2	POU6F2-F	AAGACGTCATCCCTGCACAG

		POU6F2-R	ACCCACCCATCTCTAAACTTCG
	ARID	ARID-F	TTGCCGTGTAGTCGTTCGAA
		ARID-R	ACTTCGCTGGGCTATTCCAC
	CCHC	CCHC-F	ATTTATTCAGTTCGGTAGTC
		CCHC-R	TTATTGTTTGTATTTGCAGATGTTG
	NUBBIN	NUBBIN-F	TGTGTGCGATTGTGGTCAGA
		NUBBIN-R	GGGGCGCCCTCGAATATAAA
	E(z)	E(z)-F	ACGATAAAAGCGCGTCAGTT
		E(z)-R	GCGGGATCTCTACACAACGT
	Tip60	Tip60-F	TGTTTTGACAAGAGCGTCGATT
		Tip60-R	TGAACTACCACGGCAAAAGC
qPCR analysis	MAP4K4	qMAP4K4-F	CATCAACTGGCTCCGTCTG
		qMAP4K4-R	TCATCTTCGGTGACATCCATC
	APN1	qAPN1-F	TCACTGAGTCCCATCCCA
		qAPN1-R	TGCCAGACGGCACATTTT
	APN3a	qAPN3a-F	GGCTACCGTTGGCTACAC
		qAPN3a-R	GCAGACATTCCTCCACTCC
	APN5	qAPN5-F	GGACGATCAGGCTGTTAA
		qAPN5-R	TTCCCGAATCTGGTTGTG
	APN6	qAPN6-F	CAGCAGGGACGGATAGAA
		qAPN6-R	ATCGGTAGCAACGAAGTTAA
	mALP	qmALP-F	GCACACACCATGACCGTAGCAG
		qmALP-R	GGCTCTTCGTGACATCG
	ABCB1	qABCB1-F	AGCGAAAGGAGATTGATAGG
		qABCB1-R	GTAATAAACTGGAAACCGAAC
	ABCC1	qABCC1-F	GGTGGTGCTCATCTGCTACCTCAT
		qABCC1-R	ATCCTGACACGCTCATCGGTTTT
	ABCC2	qABCC2-F	AGTCTTGGCACGCAAACGG
		qABCC2-R	CGAACAGACGCATGAAGGACAT

	ABCC3	qABCC3-F	TCAACCGCTTCACCAAGGACAT
		qABCC3-R	CGGCGTTCAGCACCAGGAT
	ABCG1	qABCG1-F	ATCTGGTGTTTCAGGCTTTAGTC
		qABCG1-R	ATCACGGTGTTCTGGCATTT
	E(z)	qE(z)-F	CGGCAAGGTCTACGACAAGT
		qE(z)-R	AGCAGTTCGGGTTGATGGAG
	Tip60	qTip60-F	ATGAGCTGCCAGAAGTGACC
		qTip60-R	TATGCCGTCCCAGCTCAATC
	RPL32	qRPL32-F	CCAATTTACCGCCCTACC
		qRPL32-R	TACCCTGTTGTCAATACCTCT
ChIP-qPCR or CUT&Tag-qPCR	$(ATGT)_n$	Upstream-F	GCTGTGAACTATCTGAGGTTGC
		Upstream-R	TGCAGAAAGAATGTACTGGGCA
		$(ATGT)_{5/34}$-F	ACCTATGTCCTTCTCCCTGCT
		$(ATGT)_{5/34}$-R	CCACTGCACGACAAGACTGA
		Downstream-F	CGGCCGCACTGAAACATATT
		Downstream-R	ACAGGACAGGTTGCACAACA
pAc5.1-TF recombinant[§]	SD	pAc-SD-F	ATGCATTCCGCCGGATATTG
		pAc-SD-R	TCAGTCTTTCACGAGCC
	CEBP	pAc-CEBP-F	ATGGAGTCCCCACAGATG
		pAc-CEBP-R	TCAGAGCGCGTGGTCGCC
	CAUP	pAc-CAUP-F	ATGTTTAACAGTAATTTA
		pAc-CAUP-R	TCAGACGGCTGACACGTA
	HOXA3	pAc-HOXA3-F	ATGTCGTCCCCAGC
		pAc-HOXA3-R	TCACAAAGATATGTAGGAGG
	EXD	pAc-EXD-F	ATGGATGATCCGA
		pAc-EXD-R	TAGACAGCGGCATC
	POU6F2	pAc-POU6F2-F	ATGTCGGAGGGCGC
		pAc-POU6F2-R	TTAGACCATGCCTTT
	ARID	pAc-ARID-F	ATGGCCAAATCA

		pAc-ARID-R	TCAGATGTTGTTCATGT
	CCHC	pAc-CCHC-F	ATGAAGTCACAGCTA
		pAc-CCHC-R	TTATTGTTTGTATTTGCAGATGT
	NUBBIN	pAc-NUBBIN-F	ATGCAGGTTCGAGGC
		pAc-NUBBIN-R	TTAATTAATGACTTC
Y1H-E(z)*	E(z)-N	E(z)-N-F	GCACCGGGACAAGGA
		E(z)-N-R	GTACTTTTCTTTAAGT
	E(z)-C	E(z)-C-F	GCTGCCGCTGCAAGGCGT
		E(z)-C-R	GTATCTGTAGTCGAAGAAC
	E(z)-FL	E(z)-FL-F	ATGAGTAAGGCCAAA
		E(z)-FL-R	TCATAAGAACTCCAT
pFastBacHT B-recombinant¶	E(z)	E(z)-F	ATGAGTAAGGCCAAA
		E(z)-R	TCATAAGAACTCCAT
	Tip60	Tip60-F	ATGAACGAAAATGAT
		Tip60-R	TCAAACACATTTGGA
pie2-EGFP-N1-recombinant¥	E(z)	E(z)-F	ATGAGTAAGGCCAAA
		E(z)-R	TCATAAGAACTCCAT
	Tip60	Tip60-F	ATGAACGAAAATGAT
		Tip60-R	TCAAACACATTTGGA
TR detection	$(ATGT)_n$	TR-Mut-F	GTAAGGGCCAACCGCTCTAGAA
		TR-Mut-R	GCTGTTGTTGTTGCAGTCCTAT
CRISPR sgRNA#	ATGT-sgRNA1	CRISPR-ATGT-F1	GAAATTAATACGACTCACTATAGG***TGTAATAATTTGAATGAATT***GTTTTAGAGCTAGAAATAGC
		CRISPR-R	AAAAGCACCGACTCGGTGCCACTTTTTCAAGTTGATAACGGACTAGCCTTATTTTAACTTGCTATTTCTAGCTCTAAAAC
	ATGT-sgRNA2	CRISPR-ATGT-F2	GAAATTAATACGACTCACTATAGG***GGATCAGTCTTGTCGTGCAG***GTTTTAGAGCTAGAAATAGC
		CRISPR-R	AAAAGCACCGACTCGGTGCCACTTTTTCAAGTTGATAACGGACTAGCCTTATTTTAACTTGCTATTTCTAGCTCTAAAAC

RNAi	E(z)	siPxE(z)	GCAAGAUACAGCUCAAGAATT UUCUUGAGCUGUAUCUUGCTT
	Tip60	siPxTip60	CACCGGGAAACGAGAUUUATT UAAAUCUCGUUUCCCGGUGTT
	Control	siNC	UUCUCCGAACGUGUCACGUTT TAAGAGGCUUGCACAGUGCA

Different restriction enzymes were used to cut the various vectors and generate linearized vectors. For pGL4.10, KpnI and BglII were used; pAC5.1, KpnI and XhoI were used; pGADT7, EcoRI and XhoI were used; pFastBacHT B and pie2-EGFP-N1, EcoRI and BamHI were used. PCR primers used to amplify the various length of promoters contained 15-bp extensions homologous to the cut vectors ends.

[†]A 15-bp sequence (TGGCCTAACTGGCCG) and a 15-bp sequence (CGCCGAGGCCAGATC) are added to the 5′-end of the forward primer (F) and reverse primer (R) for construction of pGL4.10 recombinants containing *ABCC2* promoter, respectively.

[§]A 15-bp sequence (AGACCCCGGATCGGG) and a 15-bp sequence (GCCCTCTAGACTCGA) are added to the 5′-end of the forward primer (F) and reverse primer (R) for construction of pAc5.1-TF recombinant plasmid, respectively.

[¶]A 15-bp sequence (GGGCGCCATGGGATC) and a 15-bp sequence (GTAGGCCTTTGAATT) are added to the 5′-end of the forward primer (F) and reverse primer (R) for construction of pFastBacHT B-Tip60/E(z) recombinant plasmid, respectively.

[¥]A 15-bp sequence (GGGCGCCATGGGATC) and a 15-bp sequence (GTAGGCCTTTGAATT) are added to the 5′-end of the forward primer (F) and reverse primer (R) for construction of pie2-EGFP-N1-Tip60/E(z) recombinant plasmid, respectively.

[*]A 15-bp sequence (GGAGGCCAGTGAATT) and a 15-bp sequence (TCATCTGCAGCTCGA) are added to the 5′-end of the forward primer (F) and reverse primer (R) for construction of pGADT7-PRC2/EZH2/E(z) recombinant plasmid, respectively.

[#]A specific oligonucleotide encoding a T7 polymerase-binding site (italicized) and two sgRNA target sequences (underlined and bold) of the promoter of *ABCC2* in the susceptible DBM1Ac-S strain were designed as the forward primer CRISPR-F, and a common oligonucleotide encoding the remaining sequences of CRISPR-F were designed as the reverse primer CRISPR-R.

Supplementary Table S6. Summary of positive proteins identified from yeast one-hybrid (Y1H) screening using $(ATGT)_{34}$ as bait. (enclosed excel file).

Supplementary Table S7. Summary of DNA pull-down and mass spectrometry (MS) results. (enclosed excel file).
